# Molecular Communication of Microbial Plant Biostimulants in the Rhizosphere Under Abiotic Stress Conditions

**DOI:** 10.3390/ijms252212424

**Published:** 2024-11-19

**Authors:** Sajid Ali, Muhammad Saeed Akhtar, Muhammad Siraj, Wajid Zaman

**Affiliations:** 1 Department of Horticulture and Life Science, Yeungnam University, Gyeongsan 38541, Republic of Korea; drsajid@yu.ac.kr; 2 Department of Chemistry, Yeungnam University, Gyeongsan 38541, Republic of Korea; msakhtar@yu.ac.kr; 3 Department of Biotechnology, Jeonbuk National University, Specialized Campus, Iksan 54896, Republic of Korea; sirajuom2@gmail.com; 4 Department of Life Sciences, Yeungnam University, Gyeongsan 38541, Republic of Korea

**Keywords:** microbial plant biostimulants, abiotic stress, plant–microbe interactions, phytohormone production, antioxidant enzyme activity, transcriptomics, proteomics, CRISPR, PGPR, mycorrhizal fungi

## Abstract

Microbial plant biostimulants offer a promising, sustainable solution for enhancing plant growth and resilience, particularly under abiotic stress conditions such as drought, salinity, extreme temperatures, and heavy metal toxicity. These biostimulants, including plant growth-promoting rhizobacteria, mycorrhizal fungi, and nitrogen-fixing bacteria, enhance plant tolerance through mechanisms such as phytohormone production, nutrient solubilization, osmotic adjustment, and antioxidant enzyme activation. Advances in genomics, metagenomics, transcriptomics, and proteomics have significantly expanded our understanding of plant–microbe molecular communication in the rhizosphere, revealing mechanisms underlying these interactions that promote stress resilience. However, challenges such as inconsistent field performance, knowledge gaps in stress-related molecular signaling, and regulatory hurdles continue to limit broader biostimulant adoption. Despite these challenges, microbial biostimulants hold significant potential for advancing agricultural sustainability, particularly amid climate change-induced stresses. Future studies and innovation, including Clustered Regularly Interspaced Short Palindromic Repeats and other molecular editing tools, should optimize biostimulant formulations and their application for diverse agro-ecological systems. This review aims to underscore current advances, challenges, and future directions in the field, advocating for a multidisciplinary approach to fully harness the potential of biostimulants in modern agriculture.

## 1. Introduction

The rhizosphere, a narrow soil zone influenced by plant roots, is a key site for interactions among plants, soil, and microorganisms. This dynamic, complex environment is shaped by biochemical processes driven by root exudates and microbial activity, which influences plant health, nutrient availability, and soil properties [1,2]. Microbial communities such as bacteria, fungi, and archaea establish mutualistic relationships with plant roots, enhancing nutrient acquisition, disease resistance, and stress tolerance [3,4]. The role of the rhizosphere in plant growth and development is well established, making it a key area for understanding plant adaptation to diverse environmental conditions [5]. Under abiotic stress conditions, including drought, salinity, and temperature extremes, the rhizosphere becomes particularly crucial, as it mediates the plant resilience to these adverse factors [6,7].

Microbial plant biostimulants, including plant growth-promoting rhizobacteria (PGPR), mycorrhizal fungi, and other beneficial microbes, are increasingly vital in sustainable agriculture. Unlike traditional fertilizers and pesticides, biostimulants do not supply direct nutrients or protection, they rather enhance natural plant processes, such as nutrient absorption, stress tolerance, and overall vigor [8]. These microbes interact with plants at the molecular level, shaping root architecture, boosting nutrient uptake, and activating plant defense mechanisms against environmental stressors. For instance, PGPR enhances nitrogen fixation (nifH) and phosphate solubilization, while mycorrhizal fungi improve water and nutrient uptake, particularly in nutrient-poor soils [9]. As the agricultural industry seeks sustainable methods to improve crop yields and minimize the environmental effects of chemical inputs, microbial biostimulants offer a promising alternative [10,11].

Plants are continually exposed to various abiotic stresses that can severely affect their growth, development, and productivity. Abiotic stresses are non-living environmental factors such as drought, salinity, extreme temperatures, and heavy metal toxicity, which challenge the physiological functions of plants [12]. These stresses disrupt essential processes such as photosynthesis, respiration, and water balance, resulting in reduced biomass, stunted growth, and yield losses [13]. Drought, for instance, limits water availability, causing wilting and reduced metabolic activity, while high salinity disrupts ion balance and osmotic potential [14], resulting in toxic effects on cellular functions. As climate change intensifies the frequency and severity of these stresses, developing effective solutions to mitigate their effect on agriculture has become a top priority [15].

In this context, microbial plant biostimulants are gaining attention for their ability to enhance plant tolerance to abiotic stresses by modulating physiological and biochemical responses [16]. For instance, certain microbes produce osmoprotectants and antioxidants that preserve the cellular integrity of plants during drought or salinity stress [17]. Furthermore, they can induce the production of stress-related hormones, such as abscisic acid, which regulates water loss and promotes root growth. By interacting with plant roots, these microorganisms significantly enhance water and nutrient uptake, improve soil structure, and boost plant resilience [18]. Microbial biostimulants potentially mitigate the detrimental effects of abiotic stress, offering a promising approach to improve crop performance, particularly under unpredictable conditions owing to climate change [19].

Molecular communication between plants and microbial biostimulants is crucial for understanding how these interactions contribute to stress tolerance. Plants and microbes exchange signals in the rhizosphere through root exudates, including sugars, amino acids, and secondary metabolites that attract beneficial microbes [20]. In response, microbes release signaling molecules such as phytohormones, volatile organic compounds (VOCs), and quorum-sensing molecules, which activate plant defense pathways and enhance stress tolerance. Understanding these molecular signaling pathways is crucial for optimizing biostimulant applications in agriculture [21]. For example, certain microbes produce auxins and cytokinins, which promote root elongation and branching, enhancing the ability of plants to absorb water and nutrients under stress [22]. Similarly, exopolysaccharides (EPS) produced by microbes improve soil aggregation, enhancing root stability and water retention in the rhizosphere [23].

**Figure 1:**
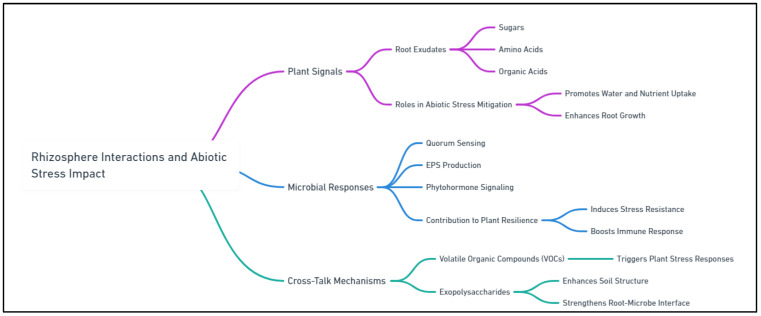
Mind map of rhizosphere interactions and abiotic stress effect.

This review aims to explore the intricate molecular mechanisms underlying interactions between microbial plant biostimulants and plants in the rhizosphere, particularly under abiotic stress conditions. It will synthesize the current knowledge on how microbial biostimulants enhance plant tolerance to drought, salinity, temperature extremes, and heavy metal toxicity. It will also examine molecular communication pathways, such as quorum sensing and hormonal signaling, that aid plant adaptation to stress. Furthermore, this review will examine how various microbial biostimulants, including PGPR, mycorrhizal fungi, and other beneficial microbes, contribute to these processes. By analyzing these molecular interactions in depth, the review highlights the potential of microbial biostimulants as a sustainable solution to the growing challenges of abiotic stress in agriculture (Figure 1). Beyond cataloging biostimulant benefits, this review aims to offer a comprehensive understanding of the molecular mechanisms underlying their efficacy. This knowledge is crucial for developing more targeted biostimulant products tailored to specific crops and stress conditions. By integrating insights from molecular biology, plant physiology, and soil microbiology, this review will provide a holistic perspective on leveraging microbial biostimulants to enhance plant resilience and productivity under environmental stress. Understanding this mechanism could benefit researchers and agricultural practitioners seeking to incorporate biostimulants into sustainable farming strategies.

## 2. Microbial Plant Biostimulants: Types and Mechanisms of Action

Microbial plant biostimulants comprise various microorganisms that enhance plant growth and stress tolerance through multiple mechanisms. Unlike traditional chemical fertilizers, biostimulants do not directly supply nutrients to plants but boost their nutrient assimilation, stress resilience, and physiological functions. These microbes work by improving root structure, enhancing water and nutrient absorption, producing growth-promoting hormones, and activating plant defense mechanisms [24]. Their effects are complex and context-dependent, relying on molecular signaling between plant roots and microbes in the rhizosphere [25]. In this section, we examine the different types of microbial biostimulants and their specific roles in promoting plant growth under normal and stressed conditions. Understanding their mechanisms of action is essential for optimizing their use in agriculture, especially in the context of sustainable farming practices.

### 2.1. Classification of Microbial Biostimulants

Microbial biostimulants can be categorized based on the types of microorganisms involved and their specific roles in the plant–soil ecosystem. The most commonly studied groups include PGPR, mycorrhizal fungi, nitrogen-fixing bacteria, and endophytic microorganisms [26]. Each group operates through distinct mechanisms, ranging from enhancing nutrient availability to inducing systemic resistance in plants [20]. Although all of these biostimulants improve plant health, they do so via different pathways that target various aspects of plant physiology and stress responses. The diversity of microbial biostimulants enables their application across a wide range of crops and environmental conditions, making them a versatile tool in modern agriculture (Table 1) [27]. Among these, mycorrhizal fungi play a key role in improving nutrient uptake by forming symbiotic associations with plant roots [28]. These fungi secrete organic acids that solubilize inorganic phosphate and other mineral nutrients, increasing their bioavailability to plants [29]. This mineral dissolution process is especially crucial in nutrient-deficient soils, where mycorrhizal fungi enhance the ability of the plant to access essential nutrients. Such interactions are vital for improving plant productivity and reducing dependence on synthetic fertilizers, positioning mycorrhizal fungi as a cornerstone of sustainable agriculture [30].

#### 2.1.1. Plant Growth-Promoting Rhizobacteria (PGPR)

PGPR are beneficial bacteria that colonize plant roots and enhance plant growth through multiple mechanisms. PGPR facilitates nutrient acquisition by solubilizing phosphorus, fixing atmospheric nitrogen (N_2_), and producing siderophores, which sequester iron for plant uptake [2,35]. Additionally, they produce phytohormones such as auxins, gibberellins, and cytokinins, which stimulate root elongation, cell division, and overall plant vigor [36]. They also contribute to biological control by suppressing plant pathogens via antibiotic production, resource competition, or by inducing systemic resistance in plants. In the rhizosphere, competition among microbial populations significantly influences the efficacy of PGPR. These beneficial bacteria must compete with pathogenic and nonpathogenic microbes for root colonization sites and scarce resources such as nutrients [37]. PGPR employs various strategies to outcompete other microbes, including producing quorum-sensing molecules and antimicrobial compounds that hinder the growth of competitors [38]. This capacity to establish dominance in the rhizosphere strengthens their role in promoting plant growth and enhancing stress tolerance [39].

PGPR can improve nutrient uptake and modulate plant defense responses; this makes them a vital component of biostimulant formulations designed to enhance crop yield and resilience [40,41]. Under abiotic stress conditions, such as drought, salinity, or temperature extremes, PGPR also helps plants adapt by modulating stress-responsive genes and pathways. For instance, some PGPR strains produce enzymes and metabolites that scavenge reactive oxygen species (ROS), thereby reducing oxidative damage in stressed plants [42]. They also alter root structure, increasing root surface area and depth to enhance water and nutrient uptake in challenging environments [43]. This multifaceted role in promoting plant growth and stress tolerance makes PGPR one of the most widely used and studied microbial biostimulants in agriculture [44].

#### 2.1.2. Mycorrhizal Fungi

Mycorrhizal fungi are symbiotic organisms that form mutualistic relationships with plant roots, greatly enhancing the ability of the plant to absorb water and nutrients, particularly phosphorus [45]. The most prevalent types of mycorrhizal fungi are arbuscular mycorrhizal fungi and ectomycorrhizal fungi, both of which boost the surface area for nutrient exchange via the specialized structures, including arbuscules and hyphal networks [46]. In addition to enhancing nutrient uptake, these fungi improve soil structure by promoting aggregation, which increases water retention and reduces soil erosion. The mycorrhizal–plant association is ancient and highly effective, playing a critical role in plant growth, especially in nutrient-poor or stressed environments [47,48].

Under abiotic stress conditions, mycorrhizal fungi support plants by improving water status and enhancing tolerance to salinity, drought, and heavy metal contamination. The extensive hyphal networks of the fungi enable plants to access water and nutrients beyond the reach of their roots, while their interaction with plant roots activates stress-responsive genes and antioxidant pathways [49,50]. Additionally, mycorrhizal fungi help modulate the hormonal balance of the plant by promoting the production of stress-related hormones such as abscisic acid, which regulates stomatal closure and aids water conservation during drought [51]. This dual role—improving resilience to environmental stresses and enhancing nutrient uptake—makes mycorrhizal fungi essential for sustainable agriculture [49].

#### 2.1.3. Nitrogen-Fixing Bacteria

Nitrogen-fixing bacteria are microorganisms that convert N_2_ into ammonia, a form of nitrogen that plants can readily assimilate. This biological nifH process is vital for plant growth, particularly in environments with limited soil nitrogen availability [52]. The most common nitrogen-fixing bacteria are rhizobia, which form symbiotic relationships with leguminous plants by colonizing root nodules and supplying the host plant with a steady nitrogen source [53]. In exchange, the plant supplies the bacteria with carbohydrates and a protective environment. Other nitrogen-fixing bacteria, including free-living diazotrophs such as *Azospirillum* and *Azotobacter*, also contribute to nifH in non-leguminous plants, although their interactions are less specialized [54,55]. 

In the context of abiotic stress, nitrogen-fixing bacteria support plant growth by sustaining nitrogen availability under nutrient-poor conditions. This is particularly significant in degraded or marginal soils with limited nitrogen [56]. Additionally, these bacteria can regulate plant stress responses by influencing gene expression involved in nitrogen metabolism and stress tolerance. Some nitrogen-fixing strains also release phytohormones that help plants maintain growth during stress, such as drought or salinity [52]. The dual role of nitrogen-fixing bacteria in providing vital nutrients and enhancing stress tolerance highlights their potential as a sustainable alternative to synthetic nitrogen fertilizers [57].

#### 2.1.4. Endophytic Microorganisms

Endophytic microorganisms, including bacteria and fungi, reside within plant tissues without harming their host. These microbes colonize the root, stem, leaf, or other tissues of the plant, providing various benefits, including enhanced nutrient uptake, improved stress tolerance, and pathogen protection [58]. Endophytes often establish highly specialized relationships with their host plants, influencing several physiological processes [59]. For example, some endophytic fungi release bioactive compounds that suppress plant pathogens or stimulate defense mechanisms, while bacterial endophytes promote plant growth by producing growth-regulating hormones or facilitating nutrient acquisition [60,61].

Endophytic microorganisms are particularly essential under abiotic stress conditions, as they help plants manage stress by modulating stress-related genes and pathways. For instance, certain endophytic fungi and bacteria produce antioxidants and osmolytes that mitigate oxidative stress induced by drought or salinity [62,63]. Additionally, endophytes can alter the root architecture of the plant and enhance water and nutrient uptake—critical in stressed environments. Their ability to reside within plant tissues and directly affect plant metabolism gives them a unique advantage in enhancing plant resilience positioning endophytic microorganisms as key targets for biostimulant research and agricultural applications [64,65].

### 2.2. General Mechanisms of Biostimulant Action

Microbial plant biostimulants promote plant growth and stress tolerance through various biochemical and physiological mechanisms [22]. These mechanisms often involve producing beneficial compounds that activate natural plant processes or enhance interactions between the plant and its surrounding environment [66]. Biostimulants can influence several key functions, including phytohormone production, nutrient solubilization, and the activation of plant defense systems [17]. Although the actions of biostimulants vary by microorganism and plant species, they share some common processes that contribute to their effectiveness [20]. These general mechanisms are essential for helping plants adapt to normal and stress conditions, maintaining productivity even in challenging environments [67].

#### 2.2.1. Phytohormone Production

**Figure 2:**
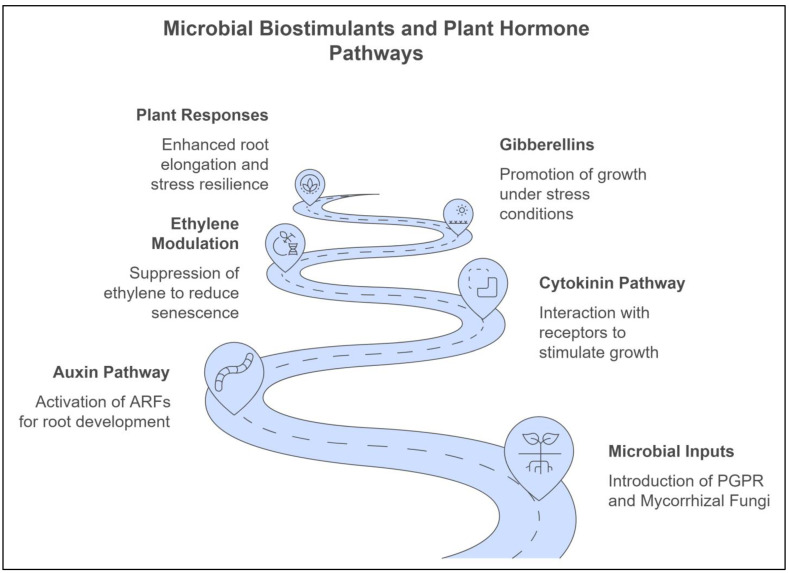
Microbial influence on phytohormone signaling pathways in plants.

One of the primary mechanisms through which microbial biostimulants function is the production of phytohormones—plant hormones that regulate key aspects of growth and development [68]. Microbial biostimulants, particularly PGPR and endophytic bacteria, can synthesize phytohormones such as auxins, cytokinins, gibberellins, and ethylene. These hormones are vital for promoting root elongation, enhancing shoot development, and controlling cell division. For instance, auxins produced by PGPR stimulate lateral root formation, increasing the root surface area and improving water and nutrient uptake [69,70]. Similarly, cytokinins modulate cell division and delay leaf senescence, helping to maintain plant vitality under stress (Figure 2) [71].

At the molecular level, microbial biostimulants influence phytohormone pathways by activating specific plant receptors and transcription factors. For instance, auxins from PGPR bind to plant receptors that activate AUXIN RESPONSE FACTORS, regulating gene expression essential for root development and elongation [72]. Microbial-produced cytokinins interact with HISTIDINE KINASE receptors on plant cell membranes, triggering response regulators that promote cell division and delay senescence, especially under stress [73]. Microbial modulation of ethylene levels also affects ETHYLENE RESPONSE FACTORS, which mediate ethylene-induced signaling to enhance stress tolerance [74]. These molecular interactions between microbial signals and plant receptors optimize growth, improve nutrient acquisition, and strengthen stress resilience, even in challenging conditions [75].

The ability of biostimulants to adjust the hormonal balance of plants is particularly valuable under abiotic stress, where natural hormone production may be disrupted. For instance, during drought stress, microbial production of auxins and gibberellins can sustain root growth, enhancing the ability of plants to access deeper water reserves [17,76]. Additionally, some biostimulants modulate ethylene levels, a hormone linked to stress responses. Biostimulants can delay premature senescence and improve plant resilience by lowering ethylene production under stress. This hormonal modulation allows plants to maintain growth and productivity despite adverse environmental conditions [77,78].

#### 2.2.2. Nutrient Solubilization and Uptake

Another key mechanism by which microbial biostimulants promote plant growth is through the solubilization and uptake of vital nutrients. Microorganisms such as PGPR and mycorrhizal fungi convert nutrients in the soil into forms that plants can readily absorb [25]. For example, phosphorus, which is often insoluble, can be made available by certain bacteria and fungi that produce organic acids and phosphatases to solubilize it [20]. Similarly, biostimulant-produced siderophores bind to iron in the soil, facilitating its uptake by plant roots. This nutrient acquisition process is crucial in nutrient-deficient soils, where traditional fertilizers may be less effective [36].

Biostimulants can also enhance nutrient uptake efficiency by increasing the root surface area through improved root architecture. Mycorrhizal fungi, for example, extend their hyphal networks beyond the root zone, enabling plants to access nutrients and water otherwise out of reach [25,79]. This is particularly beneficial under stress conditions such as drought or salinity, where nutrient availability is low. In saline soils, microbial biostimulants help plants maintain ionic balance by facilitating the uptake of key nutrients such as potassium (K^+^) while reducing the absorption of toxic ions such as sodium (Na^+^) [50]. Biostimulants enhance plant growth and also improve the efficiency of nutrient use by improving nutrient solubilization and uptake, reducing reliance on chemical fertilizers [80].

#### 2.2.3. Induced Systemic Resistance (ISR)

Induced Systemic Resistance (ISR) is a key mechanism through which microbial biostimulants enhance plant defense against pathogens and abiotic stresses. ISR involves the activation of the immune system of the plant by beneficial microorganisms, such as PGPR and certain mycorrhizal fungi. This primes the plant to respond more effectively to future pathogen attacks [81]. Unlike direct defense mechanisms, which produce antimicrobial compounds, ISR enhances systemic immunity by upregulating the expression of defense-related genes and proteins [82].

When specific biostimulants colonize plants, they undergo molecular changes that boost their ability to combat stress or infection [83]. For example, biostimulants can stimulate the production of pathogenesis-related proteins, enzymes involved in cell wall reinforcement, and antioxidants that neutralize harmful ROS [84]. These changes do not harm the plant; instead, they prime it to mount a faster and more effective defense when confronted with threats such as pathogens or environmental stress [85]. Beyond pathogen resistance, ISR improves plant tolerance to abiotic stresses such as drought, salinity, and extreme temperatures. The defense pathways activated by biostimulants often overlap with those involved in stress responses, making them effective in both domains [17,86]. However, plants often face trade-offs between growth and stress responses. For instance, stress-response activation, such as abscisic acid (ABA)-mediated stomatal closure during drought, can conserve water but also minimize photosynthesis and overall growth [87]. Biostimulants help mitigate these trade-offs by regulating hormonal balance and activating stress tolerance mechanisms without fully inhibiting growth-related processes [88]. For example, PGPR can regulate ABA and cytokinin levels, optimizing the balance between defense and growth under stress conditions. This modulation enables plants to withstand abiotic stresses while maintaining productivity [72].

ISR offers a sustainable approach to plant protection, leveraging the innate immune system of the plant, thus reducing reliance on chemical pesticides and fungicides. By boosting natural defenses, microbial biostimulants provide an eco-friendly solution to managing biotic and abiotic stresses in agriculture [89]. This makes ISR an invaluable tool in modern farming, particularly within integrated pest management and organic agriculture, where minimizing chemical inputs is a priority [90].

## 3. Abiotic Stress Conditions in Plants

Abiotic stress refers to the adverse effects of nonliving environmental factors—such as extreme temperatures, drought, salinity, and heavy metal toxicity—on plant growth and productivity [91]. These conditions can severely disrupt plant metabolism, development, and yield, causing substantial agricultural losses, particularly in areas increasingly affected by climate change [92]. In contrast to biotic stresses caused by living organisms such as pathogens or pests, abiotic stresses stem from unfavorable physical and chemical factors in the environment of the plant [93]. As these stressors become more common due to environmental degradation and climatic shifts, developing strategies to enhance plant resilience and adaptation is essential [94]. Microbial biostimulants offer a promising solution by boosting the tolerance of plants to abiotic stresses through improved root growth, stress hormone regulation, and increased nutrient and water uptake [95].

### 3.1. Types of Abiotic Stresses

Abiotic stresses vary in form depending on environmental conditions and the specific stressor involved. Common and harmful abiotic stresses in agriculture include drought, salinity, extreme temperatures, and heavy metal toxicity [96]. These stresses can act individually or together, compounding their adverse effects on plant physiology and productivity. Although each type of abiotic stress uniquely affects plants, they often disrupt similar pathways, such as water balance, nutrient uptake, and metabolic processes [96]. Understanding these distinct types of abiotic stresses and how they influence plants is critical for developing effective mitigation strategies, including the use of microbial biostimulants [17].

#### 3.1.1. Drought

Drought is among the most significant abiotic stresses on plant growth, especially in arid and semi-arid regions. It occurs when soil water availability falls short of the needs of plants, decreasing water potential and creating a severe internal water deficit [6,97]. Drought stress impairs key physiological functions such as photosynthesis, respiration, and transpiration, resulting in reduced biomass production and, in extreme cases, plant death. Symptoms of drought stress in plants include wilting, smaller leaves, and stunted growth as they struggle to preserve water. At the molecular level, drought induces the buildup of ROS, which can damage cellular structures through oxidative stress [97,98].

Plants have evolved various strategies to withstand drought, including stomatal closure to limit water loss, altering root architecture to investigate deeper soil layers, and producing osmoprotectants such as proline to maintain cell turgor [99]. Microbial biostimulants, particularly those that boost root growth and water uptake, can support plants under drought conditions by promoting water absorption efficiency and reducing oxidative stress through antioxidant production [100].

#### 3.1.2. Salinity

Salinity stress occurs when excessive soluble salts, such as sodium chloride, accumulate in the soil, leading to osmotic stress and ion toxicity in plants. Elevated salinity levels disrupt the ionic and osmotic balance within plant cells, impairing water uptake and causing ion imbalances that are toxic to plant tissues [101,102]. This stress inhibits photosynthesis, reduces nutrient uptake, and results in the accumulation of harmful ions such as Na^+^ and chloride (Cl^−^) in plant cells. Consequently, plants exhibit symptoms such as chlorosis, stunted growth, and leaf senescence [103].

In response to salinity stress, plants activate various defense mechanisms, including selective uptake of K^+^ over Na^+^, synthesis of osmolytes, and activation of antioxidant enzymes to detoxify ROS [104]. Microbial biostimulants, especially those that regulate ionic balance and enhance root function, can bolster plant tolerance to salinity by increasing the ability of the plant to exclude or compartmentalize toxic ions, thereby safeguarding cellular structures and preserving physiological functions [105].

#### 3.1.3. Extreme Temperatures (Heat and Cold)

Extreme temperatures (excessive heat or cold) cause significant stress to plants by disrupting metabolic processes. High temperatures accelerate respiration and transpiration rates, resulting in water loss, protein denaturation, and decreased photosynthetic efficiency [106,107]. In contrast, cold stress damages cellular membranes, inhibits enzyme activity, and hampers nutrient and water uptake. Heat and cold stresses induce oxidative stress owing to the overproduction of ROS, leading to cellular damage [108].

Plants respond to temperature extremes by producing heat-shock proteins (HSPs) and antifreeze proteins during heat and cold stresses, respectively. These proteins stabilize cellular structures and mitigate temperature-induced damage [109]. Microbial biostimulants boost plant resilience to extreme temperatures by enhancing their antioxidant capacity and promoting the synthesis of stress-related proteins [110].

#### 3.1.4. Heavy Metal Toxicity

Heavy metal toxicity occurs when plants are exposed to elevated levels of metals such as cadmium (Cd), lead (Pb), mercury, and arsenic (As) in the soil [111]. These metals interfere with essential biochemical processes, inhibiting enzyme activities, disrupting photosynthesis, and causing oxidative damage to cellular structures [112]. As a result, plants often exhibited stunted growth, chlorosis, and tissue necrosis [113].

To mitigate heavy metal stress, plants employ various strategies, including metal chelation by organic acids and activation of antioxidant defense systems [114]. Microbial biostimulants, particularly those that produce metal-binding compounds or enhance the detoxification pathways, can reduce heavy metal accumulation in plants [115]. Additionally, these microbes improve soil health by stabilizing heavy metals, reducing their bioavailability, and lowering the overall toxicity in the rhizosphere [116].

### 3.2. Effect of Abiotic Stress on Plant Growth and Development

Abiotic stress significantly influences plant growth and development, often resulting in reduced biomass, impaired reproductive success, and lower crop yields. Under stress conditions, plants redirect energy and resources away from growth to activate the stress response, such as synthesizing protective compounds and repairing damaged tissues (Table 2) [92]. For instance, drought stress limits leaf expansion and stem elongation, whereas salinity impedes root and shoot growth due to ion toxicity and osmotic imbalances. Extreme temperatures disrupt enzyme function and membrane integrity, and heavy metal toxicity hinders nutrient uptake and photosynthetic efficiency [15].

At the molecular level, abiotic stress activates stress-responsive genes that regulate diverse defense pathways. However, prolonged exposure can overwhelm these mechanisms, leading to irreversible damage and cell death [117]. The severity and duration of the stress, as well as the plant species and its intrinsic tolerance, determine the overall effect. Microbial biostimulants help mitigate these effects by enhancing stress tolerance, improving water and nutrient uptake, and modulating stress-responsive pathways [118].

**Table 2:** Effect of abiotic stress conditions on key physiological processes in plants.

Stress Type	Photosynthesis	Respiration	Water Uptake	References
Drought	Reduced due to stomatal closure, chlorophyll degradation	Decreased, may increase in severe cases due to energy demands for repair	Decreased, impaired root function	[119,120]
Salinity	Lowered due to osmotic stress and ion toxicity	Altered; can increase to manage ion toxicity	Impaired due to osmotic imbalance	[121,122]
High Temperature	Reduced by heat-induced chlorophyll damage, protein denaturation	Increased initially to counter heat stress, may decrease in severe conditions	Often unchanged but can reduce due to root damage	[123,124]
Low Temperature	Slowed due to enzyme inactivation	Decreased as metabolic processes slow down	Decreased due to viscosity of water in roots and soil	[125,126]
Heavy Metal Stress	Reduced by chloroplast damage and oxidative stress	Altered; increased due to reactive oxygen species (ROS) management	Decreased root growth inhibition	[127,128]

### 3.3. Rhizosphere Dynamics Under Abiotic Stress

The rhizosphere, the interface between plant roots and the surrounding soil, plays a vital role in plant adaptation to abiotic stress. Stress conditions, such as drought or salinity, can significantly alter the composition and activity of microbial communities in the rhizosphere [129], often reducing microbial diversity and function due to unfavorable soil conditions. However, certain beneficial microbes, such as PGPR and mycorrhizal fungi, can thrive under stress, providing essential services to plants. These include improving water and nutrient uptake, producing stress-related hormones, and enhancing root growth [130].

Abiotic stress also influences root exudation patterns, modifying the types and quantities of organic compounds released into the rhizosphere. These exudates act as signals that attract beneficial microbes, fostering symbiotic relationships [130]. Microbial biostimulants can further modify rhizosphere dynamics by improving soil structure, boosting nutrient cycling, and reducing the bioavailability of harmful compounds, such as heavy metals. The interaction between plants and microbial communities in the rhizosphere is dynamic, particularly under abiotic stress, and is crucial for determining the ability of a plant to survive and thrive in harsh environments. By improving rhizosphere dynamics, microbial biostimulants offer a promising strategy to enhance plant resilience to abiotic stresses in agricultural systems [129].

## 4. Molecular Communication in the Rhizosphere

**Figure 3:**
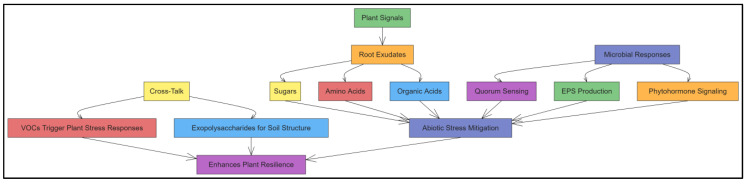
Flowchart of molecular signaling events in the rhizosphere.

The rhizosphere is a dynamic environment where plant roots and microbial communities engage in complex molecular communication crucial for establishing mutualistic relationships, particularly under environmental stress. Plants release diverse chemical signals into the rhizosphere, which attract beneficial microorganisms, including bacteria and fungi [131]. In response, these microorganisms secrete signaling molecules that enhance plant growth, development, and stress tolerance. Understanding the molecular mechanisms underlying plant–microbe interactions in the rhizosphere is essential for optimizing microbial biostimulant use in agriculture. This communication involves various signaling compounds, including root exudates, quorum-sensing molecules, and phytohormones, that collectively regulate plant–microbe interactions (Figure 3) [132].

### 4.1. Plant–Microbe Signaling in the Rhizosphere

Plant–microbe signaling in the rhizosphere is a highly coordinated process involving the exchange of chemical signals between plant roots and microbial communities. These signals are mainly transmitted via root exudates, which are produced by plant roots and act as attractants or repellents for microbes [133]. In response to these signals, microbes produce signaling molecules that trigger certain physiological responses in plants, such as increased nutrient uptake, growth promotion, or stress resistance. The effectiveness of this interaction relies on whether plants and microbes can recognize and respond to these molecular signals, with signal transduction pathways playing a crucial role in the interaction [134].

#### 4.1.1. Root Exudates: Composition and Function

Root exudates are complex mixtures of organic compounds—including sugars, amino acids, lipids, organic acids, phenolics, and secondary metabolites—secreted by plant roots into the rhizosphere. These compounds play multiple roles, such as attracting beneficial microbes, repelling pathogens, and modifying soil chemistry to enhance nutrient availability [135]. The composition of exudates differs across plant species and can vary with environmental conditions and the developmental stage of the plant. For example, under nutrient-deficient conditions, plants may release more organic acids to solubilize inaccessible nutrients such as phosphorus, whereas, under biotic stress, they may produce antimicrobial compounds to deter pathogens [136].

Root exudates are crucial for shaping the microbial community in the rhizosphere. Beneficial microbes, such as PGPR, are often attracted to the sugars and amino acids in exudates, allowing them to colonize the root surface and form symbiotic relationships [135]. In response to these exudates, microbes can produce growth-promoting compounds or trigger plant defense pathways, enhancing the ability of the plant to cope with abiotic stress. Therefore, root exudates are essential mediators of plant–microbe communication, influencing microbial activity and plant health [137].

#### 4.1.2. Signal Transduction Pathways in Plants and Microbes

Signal transduction pathways are molecular mechanisms through which plants and microbes perceive and respond to external signals. In plants, these pathways often involve receptor proteins on the plant cell surface that recognize microbial signals and activate intracellular signaling cascades, leading to specific physiological responses. For example, some PGPR produce lipochitooligosaccharides that bind to plant receptors, initiating root nodulation in legumes. Similarly, plants detect microbial-associated molecular patterns, such as flagellin or chitin, which activate defense-related pathways to guard against pathogens [138].

In microbes, signal transduction involves detecting plant-derived compounds through membrane-bound receptors or quorum-sensing systems that control microbial behavior in response to the plant environment. These pathways are essential for coordinating microbial activities, such as biofilm formation, motility, and the production of beneficial compounds. Understanding the molecular specifics of these pathways can help researchers develop strategies to enhance plant–microbe interactions, ultimately promoting plant health and resilience through microbial biostimulants [139].

### 4.2. Role of Quorum Sensing in Microbial Populations

Quorum sensing is a bacterial communication mechanism that enables microbes to coordinate behavior based on population density. This process involves producing and detecting small signaling molecules called autoinducers, which accumulate in the environment as the microbial population increases. When the concentration of autoinducers reaches a significant threshold, it triggers a coordinated response across the bacterial community, such as biofilm formation, production of virulence factors, or antibiotic synthesis [140].

In the rhizosphere, quorum sensing is essential for regulating microbial interactions with plants. For instance, some plant growth-promoting bacteria (PGPB) use quorum sensing to control the production of compounds that enhance plant growth, such as phytohormones and siderophores [141]. Additionally, quorum sensing can affect biofilm formation, which helps microbes colonize plant roots more effectively and protects them from environmental stressors. Manipulating quorum-sensing systems may allow scientists to amplify the beneficial effects of microbial biostimulants, promoting plant health and resilience in agricultural systems [142].

### 4.3. Molecular Cross-Talk Between Microbes and Plants

The molecular cross-talk between plants and microbes in the rhizosphere is mediated by various signaling compounds that regulate plant growth, stress tolerance, and defense responses. These signals, including phytohormones, VOCs, and EPS, allow microbes to interact with plants and influence their physiological processes. This cross-talk is crucial for establishing beneficial plant–microbe relationships, enabling both partners to coordinate their responses to environmental cues [143,144].

#### 4.3.1. Hormonal Signaling (Auxins, Cytokinins, and Ethylene)

Phytohormones play a key role in the molecular communication between plants and microbes. Microbial biostimulants, such as PGPR and endophytic bacteria, can produce phytohormones, such as auxins, cytokinins, and ethylene, which modulate plant growth and development [145]. For example, auxins produced by PGPR stimulate root elongation and branching, enhancing the ability of the plant to absorb water and nutrients. In contrast, cytokinins promote cell division and delay leaf senescence, improving overall plant vigor. Although ethylene is typically associated with stress responses, microbes can modulate its production to mitigate its negative effects under stressful conditions [66].

Microbial regulation of phytohormone levels is especially crucial under abiotic stress, as these hormones help plants adapt to adverse conditions. By producing or modulating phytohormones, microbial biostimulants support plant growth and productivity even in challenging environments, such as drought, salinity, or extreme temperature [20].

#### 4.3.2. Microbial Production of Volatile Organic Compounds (VOCs)

VOCs are small, diffusible molecules produced by microbes that can affect plant growth and stress responses even at low concentrations. These compounds, including alcohols, ketones, and terpenes, act as signaling molecules in plant–microbe interactions. Certain PGPR and fungi produce VOCs that boost plant growth by enhancing root development, increasing photosynthesis, or activating plant defense pathways [146].

Additionally, VOCs play a key role in improving plant tolerance to abiotic stress. For instance, VOCs from beneficial microbes upregulate stress-responsive genes in plants, including those involved in the antioxidant defense system. This aids plants in managing oxidative stress caused by drought or salinity. The ability of VOCs to mediate long-distance signaling between plants and microbes positions them as valuable components in the development of biostimulants that enhance plant resilience in stressed environments [147].

#### 4.3.3. Exopolysaccharides (EPS) in Stress Tolerance

EPS are high-molecular-weight polysaccharides secreted by microbes into their surrounding environment. These compounds are crucial for microbial biofilm formation, enabling microbes to adhere to plant roots and create protective barriers against environmental stress. EPS also improves soil structure by promoting aggregation, which enhances water retention and root penetration. This is especially beneficial in drought or saline conditions, where water availability is limited, and soil structure is degraded [148].

Beyond their structural role, EPS protects plants from stress by reducing the accumulation of toxic ions, including Na+ in saline soils, and scavenging ROS, which can damage plants under stress. Microbial biostimulants that produce EPS can enhance plant tolerance to abiotic stress by improving the physical and chemical environment around the roots, thus creating a more favorable growth niche. As a result, EPS-producing microbes are valuable components in biostimulant formulations aimed at improving plant resilience in challenging environments [149].

## 5. Microbial Plant Biostimulants and Abiotic Stress Mitigation

Abiotic stress is a major of reduced crop productivity worldwide, with plants facing diverse environmental challenges, including drought, salinity, extreme temperatures, and heavy metal toxicity. Microbial plant biostimulants offer a promising strategy for mitigating the negative effects of these stresses on plant growth and development [91]. By enhancing the natural defense mechanisms of the plant, improving nutrient and water uptake, and modulating stress-responsive pathways, microbial biostimulants help plants survive and thrive under adverse conditions. In this section, we investigate the mechanisms by which biostimulants enhance plant tolerance to abiotic stress and review case studies that demonstrate their effectiveness across various stress conditions [150].

### 5.1. Mechanisms by Which Biostimulants Enhance Abiotic Stress Tolerance

Microbial biostimulants alleviate the effects of abiotic stress through different well-documented mechanisms (Table 3), including osmotic adjustment via microbial metabolites, enhanced antioxidant enzyme activity, and improved water and nutrient uptake. These mechanisms work synergistically to protect plants from stress-induced damage and enhance their ability to recover from unfavorable conditions. Biostimulants support plant growth and productivity, even under severe environmental stress, by optimizing these processes.

#### 5.1.1. Osmotic Adjustment via Microbial Metabolites

Osmotic adjustment is a critical mechanism by which plants manage abiotic stress, particularly under drought and salinity. In these conditions, limited water availability induces osmotic stress, disrupting cellular water balance and impairing physiological functions [155]. Certain microbial biostimulants produce metabolites, such as osmolytes—including proline, glycine betaine, and trehalose—which help plants maintain osmotic balance. These compounds act as compatible solutes, stabilizing cellular structures and reducing water loss during stress [156].

Microbial production of osmolytes can also stimulate the synthesis of these compounds, further improving the ability of the plant to withstand osmotic stress. For example, plants inoculated with PGPR that produce osmoprotectants exhibit improved drought and salinity tolerance. These microbes help regulate water uptake and retention at the cellular level. This osmotic adjustment mechanism allows plants to maintain turgor pressure, preventing wilting and supporting growth and development under water-limited conditions [157,158].

#### 5.1.2. Enhanced Antioxidant Enzyme Activity

Abiotic stress triggers the overproduction of ROS in plants, which can lead to oxidative damage to cellular components, such as lipids, proteins, and nucleic acids. To combat this, plants activate antioxidant defense systems, including enzymes such as superoxide dismutase (SOD), catalase (CAT), and peroxidases, which detoxify ROS and hinder cellular damage. Microbial biostimulants can promote this antioxidant response by directly producing antioxidant compounds or stimulating the antioxidant enzyme system of the plant [159,160].

Studies report that plants treated with specific biostimulants show increased antioxidant enzyme activity, reducing oxidative stress under drought, salinity, and extreme temperature conditions. For example, mycorrhizal fungi and PGPR increase the activity of SOD and CAT, thereby mitigating oxidative stress effects. By enhancing the antioxidant capacity of plants, biostimulants not only protect against ROS-induced damage but also improve overall stress resilience [161].

#### 5.1.3. Improvement in Water and Nutrient Uptake

One of the significant ways that microbial biostimulants help plants cope with abiotic stress is by enhancing water and nutrient uptake. Under drought or saline conditions, water and nutrient availability are often limited, severely affecting plant growth. Mycorrhizal fungi, in particular, play a crucial role by extending their hyphal networks into the soil, allowing plants to access water beyond the reach of their roots. Additionally, these fungi improve nutrient uptake, especially phosphorus, which is vital for energy metabolism and stress tolerance [162].

In addition to mycorrhizal fungi, PGPR and other beneficial microbes enhance nutrient uptake by solubilizing otherwise unavailable nutrients, such as phosphorus, and by producing siderophores that bind to iron, making it more accessible to plants. These biostimulants also stimulate the growth of more extensive root systems, increasing the ability of the plant to absorb water and nutrients. By improving water and nutrient acquisition, biostimulants help maintain metabolic processes and support plant growth, even under stressful environmental conditions [78].

### 5.2. Case Studies of Microbial Biostimulants Under Specific Stress Conditions

Numerous case studies show the effectiveness of microbial biostimulants in mitigating abiotic stress across various stress conditions. These studies emphasize the role of biostimulants in enhancing plant tolerance to drought, salinity, heat, and heavy metal toxicity and provide insights into the mechanisms by which these microbes exert their beneficial effects [163].

#### 5.2.1. Drought Stress: Role of PGPR and Mycorrhizal Fungi

Drought stress is a prevalent abiotic challenge for crops, and microbial biostimulants show significant potential in enhancing drought tolerance. PGPR, such as *Azospirillum* and *Bacillus* species, along with mycorrhizal fungi, have been well studied for their role in improving plant resilience to drought. These microbes aid plants in coping with water scarcity by promoting root growth, enhancing water uptake, and stimulating osmoprotectant production [164].

For instance, mycorrhizal fungi form symbiotic connections with plant roots, extending their hyphae into deeper soil layers to access additional water. This increased water absorption capacity helps plants maintain hydration and avoid wilting under drought conditions. Conversely, PGPR can produce auxins that stimulate root growth, resulting in larger root systems capable of more efficient water access. A study shows that crops treated with PGPR and mycorrhizal fungi exhibit improved drought tolerance, higher biomass production, and better yield under water-limited conditions than that of untreated plants [165].

#### 5.2.2. Salinity Stress: Biostimulant-Mediated Ionic Balance

Salinity stress threatens plant growth by disrupting ionic balance and causing osmotic stress. Excessive soil salt leads to the buildup of toxic ions, such as Na^+^ and Cl^−^, which interfere with essential physiological processes. Microbial biostimulants, particularly PGPR and specific halophilic fungi, help plants manage salinity stress by promoting ionic balance and improving nutrient uptake [166].

A primary mechanism by which biostimulants mitigate salinity stress is by promoting the selective uptake of essential ions, such as K^+^, while excluding or compartmentalizing toxic ions, such as Na^+^. PGPR, including *Pseudomonas* and *Bacillus* species, produces enzymes that regulate ionic transporters in plants, helping them maintain ion homeostasis under saline conditions [167,168]. Mycorrhizal fungi also support ionic balance by enhancing the ability of the plant to exclude toxic ions from root tissues, thus protecting cellular structures from salt-induced damage. Consequently, plants treated with microbial biostimulants demonstrate higher salt tolerance, better growth, and improved nutrient status under saline conditions [169].

#### 5.2.3. Heat Stress: Alleviation via Microbial VOCs

Heat stress can substantially reduce crop yields by damaging cellular structures, disrupting metabolic processes, and accelerating water loss through transpiration. Microbial biostimulants help mitigate heat stress via various mechanisms, including the production of VOCs that modulate plant stress responses. VOCs produced by specific PGPR and fungi enhance plant heat tolerance by regulating stress-responsive genes and bolstering antioxidant defense systems [43].

For example, VOCs such as 2,3-butanediol and acetoin, produced by *Bacillus* species and related genera, trigger the expression of HSPs and other stress-related proteins in plants, helping them endure elevated temperatures. These VOCs also stimulate antioxidant production, protecting plants from heat-induced oxidative stress. A study shows that plants treated with VOC-producing biostimulants exhibit better growth, higher chlorophyll content, and improved photosynthetic efficiency under heat-stress conditions [170].

#### 5.2.4. Heavy Metal Stress: Microbial Detoxification Mechanisms

Heavy metal toxicity in contaminated soils is a significant issue, as metals such as Cd, Pb, and As can accumulate to toxic levels, hindering plant growth and development. Microbial biostimulants, including metal-resistant bacteria and fungi, help plants tolerate heavy metal stress by enhancing metal detoxification and sequestration. These microbes produce metal-binding compounds such as siderophores, EPS, organic acids, and metallothioneins, which chelate toxic metals and lower their bioavailability in the soil [171].

For instance, PGPR such as *Pseudomonas* and *Bacillus* species produce siderophores that bind to heavy metals, preventing their uptake by plants and decreasing their toxicity. Additionally, some microbes stimulate the production of phytochelatins in plants, which sequester heavy metals in vacuoles, thereby minimizing their harmful effects. A study shows that plants treated with metal-detoxifying biostimulants exhibit greater tolerance to heavy metal stress, improved growth, and lower metal accumulation in their tissues [172].

## 6. Genomics and Molecular Tools in Biostimulant Research

**Figure 4:**
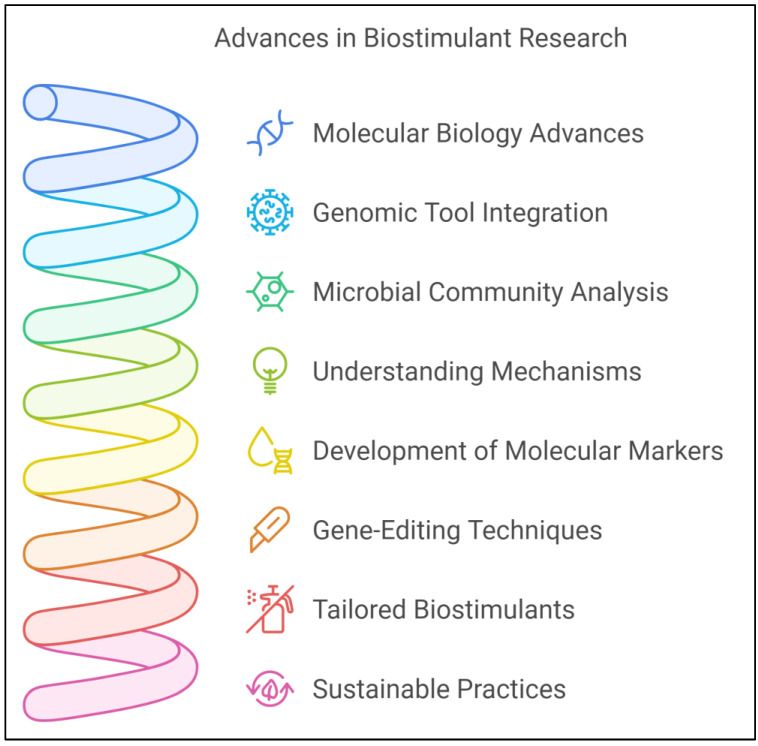
Genomic and molecular approaches in biostimulant research.

Advances in molecular biology, genomics, and related technologies have accelerated the study and application of microbial plant biostimulants. These technologies allow researchers to analyze rhizosphere microbial communities with unprecedented precision, identifying specific genes, proteins, and metabolic pathways that contribute to plant growth promotion and stress tolerance [173]. The use of genomic and molecular tools has transformed biostimulant research by providing insights into the microbial mechanisms that enhance plant resilience to abiotic stresses (Figure 4). Additionally, the development of molecular markers and gene-editing techniques offers new opportunities to optimize biostimulant efficacy and ensure their targeted application in agriculture [174].

### 6.1. Advances in Genomics and Metagenomics for Studying Microbial Biostimulants

Genomic technologies, particularly next-generation sequencing, significantly advance our understanding of microbial biostimulants by enabling detailed analysis of microbial genomes and community structures. Whole-genome sequencing allows researchers to detect the genes responsible for essential functions, such as nutrient solubilization, phytohormone production, and stress tolerance in individual microbial species [175]. By characterizing the genomes of PGPB and mycorrhizal fungi, researchers can elucidate the molecular basis of their beneficial effects on plants. For instance, sequencing the genomes of *Rhizobium* species reveals key genes involved in nifH and plant–microbe signaling [176].

Beyond genomics, metagenomics has become a powerful tool for examining complex microbial communities in the rhizosphere. Unlike traditional methods that require microbial culturing, metagenomics enables the direct analysis of microbial DNA from environmental samples, offering a comprehensive overview of the microbial diversity and functional potential in the rhizosphere [177]. This approach is crucial in discovering novel microbial species and metabolic pathways that support plant growth and stress resilience. Through metagenomic analysis, researchers can also examine how microbial communities adapt to abiotic stress conditions, such as drought or salinity, and identify which microbial taxa are most effective at enhancing plant tolerance to these stresses [178].

### 6.2. Molecular Markers for Tracking Biostimulants in the Rhizosphere

Molecular markers and specific DNA sequences are critical tools for tracking and monitoring the presence and activity of microbial biostimulants in the rhizosphere. These markers help researchers understand the persistence, colonization patterns, and ecological effects of biostimulants in agricultural soils [179]. One of the most commonly used molecular markers in biostimulant research is the 16S ribosomal RNA (rRNA) gene, a highly conserved sequence among bacterial species. Using 16S rRNA gene sequencing, researchers can identify and quantify microbial species in the rhizosphere before and after biostimulant application [180].

In addition to taxonomic markers such as 16S rRNA, functional gene markers are employed to track specific biostimulant activities in the rhizosphere. For example, genes involved in nifH, phosphate solubilization, and hormone production (for ACC deaminase) can be monitored to assess the functional effect of biostimulants on plant growth and stress responses. These markers provide valuable insights into how biostimulants interact with native microbial communities, their persistence over time, and their influence on plant physiology under stress conditions [181].

The use of fluorescent markers and reporter genes, such as green fluorescent protein, further enhances the real-time tracking of biostimulants. These markers enable researchers to visualize microbial colonization of plant roots and monitor the spatial distribution of biostimulants in the rhizosphere. By integrating these molecular tools, researchers can optimize microbial biostimulant use in agriculture, enhancing their effectiveness and sustainability [182].

### 6.3. Transcriptomics and Proteomics: Insights into Stress Responses

Transcriptomics and proteomics offer valuable insights into the molecular responses of plants and microbes to abiotic stress. Transcriptomics involves studying gene expression by analyzing messenger RNA transcripts, while proteomics focuses on identifying and quantifying proteins produced by cells. Together, these methods clarify how microbial biostimulants influence plant stress responses at the molecular level [183].

Using transcriptomics, researchers can observe how the biostimulants alter gene expression patterns in plants, particularly for genes associated with stress tolerance, growth regulation, and nutrient uptake [184]. For instance, transcriptomic studies show that plants treated with PGPR exhibit upregulation of genes linked to antioxidant defense, osmotic regulation, and hormone signaling under drought stress. Transcriptomic analysis of microbial biostimulants also reveals microbial genes activated in response to plant exudates or environmental stressors, offering insights into the mechanisms that enhance plant resilience [185,186].

Proteomics complements transcriptomics by examining the functional proteins involved in stress responses. Although transcriptomic data show which genes are transcribed, proteomics reveals which proteins are actively produced and functioning within the cells [187]. Proteomic studies demonstrate that biostimulant-treated plants increase levels of stress-related proteins, such as HSPs, antioxidant enzymes, and ion transporters, which help alleviate the effects of abiotic stress. By integrating transcriptomics and proteomics, researchers can gain a holistic view of the molecular networks underlying biostimulant-mediated stress tolerance [188].

### 6.4. CRISPR and Other Molecular Editing Tools in Biostimulant Engineering

Clustered Regularly Interspaced Short Palindromic Repeats (CRISPR) and other gene-editing tools have transformed biotechnology, offering precise and efficient genome modifications in various organisms [189]. In biostimulant research, CRISPR shows significant potential for engineering microbial strains with enhanced properties for promoting plant growth and mitigating stress. By targeting specific genes involved in nutrient solubilization, hormone production, or stress resilience, scientists can create “designer” biostimulants tailored to specific crops and environmental conditions [190].

A key application of CRISPR in biostimulant research is modifying microbial genomes to improve their stress tolerance and colonization abilities. For example, CRISPR can be used to delete genes that reduce the ability of the microbe to survive under drought or salinity or insert genes that promote beneficial metabolite production, such as osmolytes or antioxidants [191]. CRISPR-based tools can also be used to optimize biostimulant–plant interactions by engineering microbes to produce more effective signaling molecules that activate plant defense pathways or improve nutrient uptake [192].

Beyond CRISPR, other molecular editing tools, such as Transcription Activator-Like Effector Nucleases and Zinc Finger Nucleases, are explored for biostimulant engineering. These tools provide alternative methods for precise gene modification, allowing researchers to refine the functions of microbial biostimulants [193]. Although engineering microbial strains hold great promise, potential trade-offs in plant–microbe interactions must be considered. For instance, the metabolic demands of engineered biostimulants could theoretically disrupt the energy balance of the plant, particularly if the microbial functions are overly active under non-stress conditions [194]. To address this, current engineering strategies focus on activating stress response mechanisms only in response to specific environmental cues, reducing the continuous energy burden. By controlling microbial gene expression contextually, engineered biostimulants are designed to support plant resilience without compromising growth or acting as weak pathogens [195]. As gene-editing technologies advance, the development of custom-engineered biostimulants will be crucial for enhancing crop productivity and sustainability in the face of growing environmental challenges [196].

In summary, the integration of genomic, transcriptomic, proteomic, and gene-editing technologies significantly expands the potential of microbial biostimulants in agriculture. These molecular approaches not only provide deeper insights into the complex interactions between plants and microbes but also facilitate the development of more effective and targeted biostimulant products [197]. As global demand for sustainable agricultural solutions grows, leveraging these cutting-edge technologies will be vital in optimizing microbial biostimulants to mitigate abiotic stress and improve crop resilience [198]. These advances in omics and synthetic biology are also transforming the landscape of microbial biostimulant research [199]. Omics technologies such as metagenomics and metabolomics allow the investigation of the specific functions and metabolic outputs of rhizosphere microbes, identifying targets for microbial enhancement [200]. Simultaneously, tools such as CRISPR enable precise genetic modifications that enhance traits such as stress tolerance and metabolite production, leading to the creation of customized ‘designer’ biostimulants [197]. Integrating these tools paves the way for next-generation biostimulants with optimized interactions, stability, and efficacy across various environmental conditions [201]. Together, omics and synthetic biology are propelling biostimulant research toward highly targeted, sustainable, and resilient agricultural solutions [202].

## 7. Challenges and Future Directions

**Figure 5:**
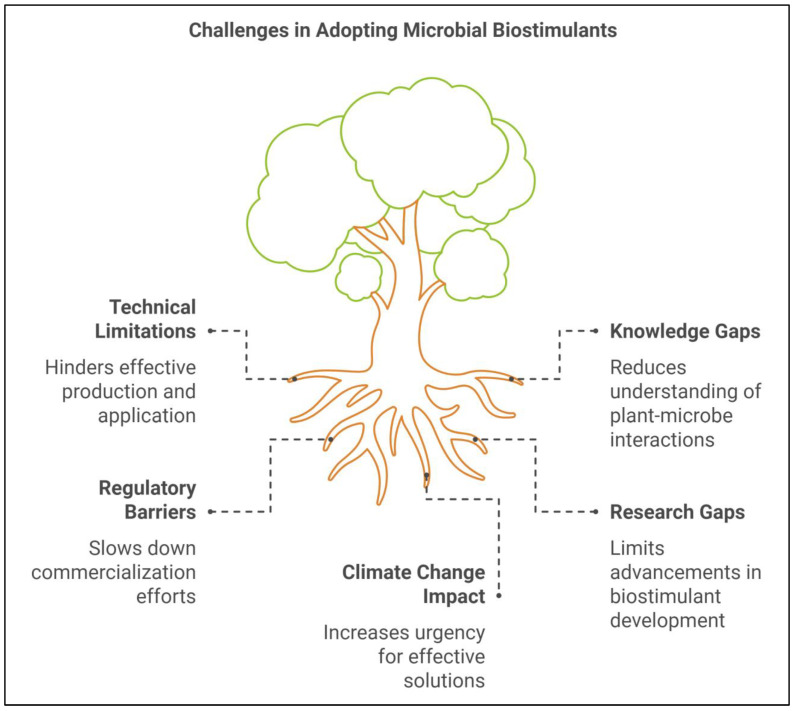
Key challenges in the use of microbial biostimulants.

**Figure 6:**
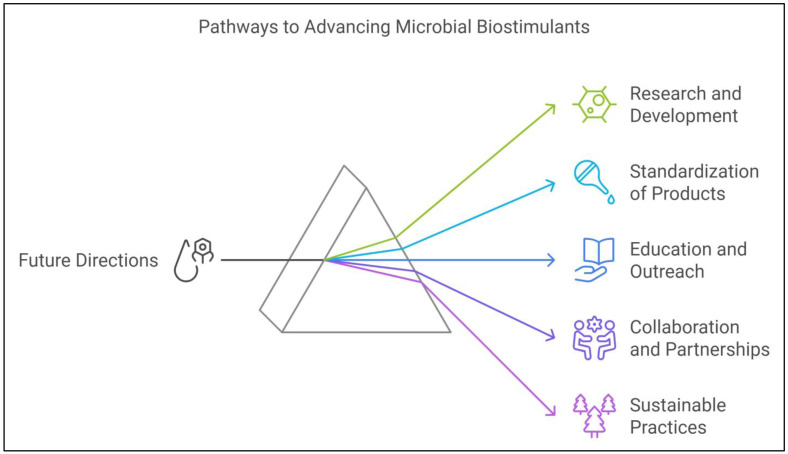
Key pathways for advancing microbial biostimulant research and applications.

The potential of microbial plant biostimulants to mitigate abiotic stress and boost agricultural productivity is widely recognized, but their widespread use faces several challenges. These include technical limitations in production and application, as well as significant knowledge gaps in understanding the mechanisms of plant–microbe interactions, particularly under stressful conditions [203]. Furthermore, regulatory and commercialization barriers hinder the broader adoption of biostimulants in conventional and sustainable agricultural practices. Despite these issues, the prospects for microbial biostimulants remain promising, especially as a critical tool for addressing climate change-induced stresses on crops [204]. In this section, we explore the current limitations and challenges linked to biostimulant use, identify research gaps, and investigate future directions for advancing the development and application of microbial biostimulants in agriculture (Figure 5 and Figure 6).

### 7.1. Current Limitations in the Application of Microbial Biostimulants

A major limitation in the use of microbial biostimulants is their inconsistent performance under field conditions. While biostimulants show promising results in controlled environments such as greenhouses and growth chambers, their efficacy can differ significantly in real-world agricultural systems [205]. This inconsistency is primarily due to the complexity of soil ecosystems, environmental factors, and the diversity of microbial communities in the rhizosphere. Factors such as soil pH, temperature, moisture, and organic matter content can all affect the survival, colonization, and activity of microbial biostimulants [27]. Additionally, communications between introduced biostimulants and native microbial populations could either enhance or inhibit the efficacy of the applied microbes, depending on the ecological context [206]. However, certain exceptions, such as *Epichloe* endophytes in grasses and nitrogen-fixing microbial associations in legumes, are relatively stable in field conditions [207]. These successful associations are attributed to the specialized, often coevolved relationships between host plants and microbes, which provide consistent benefits even in different environments [208]. These examples underscore the potential of targeted biostimulants that leverage certain plant–microbe interactions to achieve reliable field performance [209].

To address site variability, targeted strategies are being developed to align biostimulants with specific environmental conditions [210]. One such approach involves creating site-specific microbial consortia that are tailored to local soil characteristics and native microbial populations. Advancements in metagenomics and soil microbiome analysis enable the precise selection of biostimulants that complement existing microbial communities, potentially enhancing their performance in the field [211]. The use of encapsulation techniques also helps protect microbial viability and regulate release rates under varying environmental conditions, enhancing stability across several pH and moisture levels. These strategies show promise in overcoming field inconsistencies and enhancing the effectiveness of biostimulants in diverse agricultural settings [212].

Another limitation is the lack of crop-specific biostimulant formulations. Most commercially available products are broad-spectrum biostimulants intended to benefit various crops [213]. However, the effectiveness of a biostimulant may vary significantly depending on the crop species, type of stress it faces, and local environmental conditions. More research is necessary to develop biostimulant formulations tailored to the specific needs of different crops and stress factors to ensure more consistent and reliable results [214].

### 7.2. Practical Application Strategies and Ecological Impact

To ensure the consistent performance of microbial biostimulants in field conditions, strategies must address environmental variability and promote stability across different ecosystems [215]. One effective approach is the use of tailored microbial consortia, which complement native soil microbial communities and enhance specific plant–microbe communications [216]. These consortia can improve the efficacy and resilience of biostimulants under varying field conditions by selecting microbial strains suited to local soil and crop conditions. Additionally, encapsulation technologies are being explored to guard microbial biostimulants, allowing for controlled release and better survival rates under challenging environmental conditions, including fluctuating soil moisture and pH [7,217].

Beyond practical application strategies, considering the ecological influence of biostimulants on local microbial ecosystems is essential. The introduction of biostimulants into the soil may interact with native microbial communities, potentially altering microbial diversity and disrupting ecological balance [218]. Although biostimulants generally enhance soil health and nutrient cycling, their long-term effects on ecosystem biodiversity require further investigation. Understanding these ecological interactions will enable researchers to develop biostimulants that not only support crop tolerance but also enhance soil biodiversity and overall ecosystem health.

### 7.3. Knowledge Gaps in Molecular Communication Under Stress

Despite significant progress in understanding the role of microbial biostimulants in plant growth and stress tolerance, knowledge gaps remain, particularly regarding the molecular communication between plants and microbes under stress conditions [66]. The molecular mechanisms through which microbes enhance plant tolerance to abiotic stress—such as the signaling pathways contributing to root–microbe interactions and the specific genes or proteins that mediate these responses—are not fully understood [219]. For example, while certain microbes are known to produce phytohormones or antioxidants that help plants withstand stress, the precise molecular mechanisms by which these compounds regulate plant stress responses remain unclear [129].

Additionally, the dynamics of plant–microbe signaling under various types of abiotic stress are not well understood. For instance, the molecular signals involved in drought tolerance may vary from those associated with salinity or temperature stress, and the communications between different signaling pathways in response to combined stressors are poorly characterized [220]. Furthermore, the role of microbial communities in the rhizosphere, including the interactions among microbial species and how these interactions influence plant stress tolerance, requires further investigation. Addressing these gaps is critical for optimizing biostimulant use and improving their efficacy under diverse environmental conditions [221].

### 7.4. Regulatory and Commercialization Challenges

The regulatory landscape for microbial biostimulants is complex and differs widely across regions, presenting significant challenges for their commercialization and widespread adoption [222]. In many countries, biostimulants are regulated under frameworks designed for fertilizers or pesticides despite their different mechanisms of action. This overlap can create barriers to market entry, as companies must navigate lengthy and costly registration processes to prove the safety and efficacy of their products [223].

Another key regulatory challenge is the lack of standardized definitions and criteria for biostimulants. Although some countries are developing specific regulations for biostimulants, considerable variability in how these products are classified and regulated exists. This lack of harmonization complicates global market expansion for companies and creates uncertainty for farmers regarding the regulatory status and effectiveness of the products they use [224].

Commercialization is further complicated by the unique nature of microbial biostimulants as living organisms. Unlike chemical inputs such as fertilizers or pesticides, microbial biostimulants face challenges related to viability and shelf life, which can affect their performance [225]. Developing formulations that maintain the long-term stability and activity of microbial biostimulants remain an ongoing industry challenge. Additionally, more extensive field trials and demonstration projects are needed to build confidence among farmers and agronomists in the reliability of microbial biostimulants as part of integrated crop management [10].

### 7.5. Future Prospects: Biostimulants as a Sustainable Solution to Climate Change-Induced Stress

Despite the challenges, the future of microbial biostimulants is promising, particularly in addressing climate change-induced stresses on agriculture. With rising global temperatures and more frequent extreme weather events, such as droughts, floods, and heat waves, the demand for sustainable approaches to boost crop resilience is increasing [226]. Microbial biostimulants represent a viable solution for mitigating climate change effects on crop production by enhancing plant tolerance to various abiotic stresses [227].

Advances in genomics, transcriptomics, and molecular biology will possibly drive the development of next-generation biostimulants, making them more targeted and effective. For example, CRISPR and other gene-editing tools could be used to engineer microbial strains with enhanced stress tolerance or colonization abilities, resulting in “designer” biostimulants tailored to specific crops and environmental conditions. Additionally, integrating biostimulants with other sustainable agricultural practices, such as precision agriculture and organic farming, could enhance their role in promoting soil health, reducing chemical inputs, and improving agricultural sustainability overall [156,228].

Moreover, investment from the public and private sectors will be essential to advance biostimulant technologies. Collaboration among academic institutions, industry, and policymakers is needed to address regulatory challenges [229], conduct large-scale field trials, and develop crop-specific formulations that maximize the effectiveness of microbial biostimulants [230]. As the agricultural sector adapts to climate change, microbial biostimulants will increasingly support global food security and sustainable farming practices [231]. Although challenges remain in developing and applying microbial biostimulants, their potential to enhance crop resilience and productivity under abiotic stress conditions positions them as key tools for sustainable agriculture. Continued research, regulatory innovation, and commercial development are essential to unlocking their full potential and facilitating widespread adoption in farming systems worldwide [232].

## 8. Conclusions

Microbial plant biostimulants show strong potential to enhance plant growth, improve nutrient uptake, and mitigate abiotic stress effects. Through various mechanisms, including phytohormone production, nutrient solubilization, osmotic adjustment, and antioxidant system enhancement, biostimulants support plant resilience against drought, salinity, extreme temperatures, and heavy metal toxicity. The integration of modern genomic and molecular tools has deepened our understanding of the specific genes, proteins, and pathways involved in plant–microbe interactions, offering new opportunities to optimize biostimulant application in agriculture. Despite these advancements, challenges such as inconsistent field performance, knowledge gaps in molecular communication, and regulatory hurdles remain, necessitating further research and development to fully realize the potential of microbial biostimulants in sustainable agriculture.

The findings have substantial practical implications for agriculture. As climate change increases the frequency and severity of abiotic stresses, adopting microbial biostimulants can provide farmers with a valuable tool to enhance crop resilience and productivity. Biostimulants offer a more sustainable alternative to conventional fertilizers and pesticides by boosting natural plant processes and reducing chemical input reliance. This supports healthier soils and ecosystems and promotes the long-term viability of agricultural systems. However, maximizing biostimulant benefits requires tailored formulations specific to various crops and stress conditions, along with extensive field trials to validate effectiveness across diverse agro-ecological environments. Developing stable, easy-to-apply biostimulant products that are compatible with current farming practices will be crucial for their widespread adoption. 

In conclusion, increased research on microbial biostimulants underscores their potential to support more sustainable and resilient agricultural systems amid global environmental challenges. However, fully realizing this potential will require a multidisciplinary approach, integrating expertise from plant biology, microbiology, soil science, genomics, and agricultural engineering. Collaboration among researchers, industry stakeholders, and policymakers is essential to overcome the current challenges, develop innovative solutions, and establish regulatory frameworks that support safe and effective biostimulant applications. Continued investment in research and development, alongside farmer education and outreach, will be crucial for integrating microbial biostimulants as a core component of modern agricultural practices.

## Figures and Tables

**Table 1:** Classification of microbial biostimulants: types, functions, target outcomes, and mechanisms of action.

Biostimulant Type	Main Functions	Target Outcomes	Mechanisms of Action	References
Plant Growth-Promoting Rhizobacteria (PGPR)	Nutrient solubilization, root development, and phytohormone production	Enhanced nutrient uptake and improved growth	Phytohormone regulation and Induced Systemic Resistance (ISR) induction	[31]
Mycorrhizal Fungi	Increased nutrient availability and stress tolerance	Improved stress resilience and plant biomass	Enhanced root colonization and P uptake	[32]
Nitrogen-Fixing Bacteria	Nitrogen fixation and antifungal activity	Improved nitrogen nutrition and disease control	Root colonization and nitrogenase enzyme	[33]
Endophytes	Disease resistance and phytohormone production	Increased resistance to pathogens	Antifungal activity and ISR induction	[34]

**Table 3:** Influence of microbial biostimulants on plant resilience to abiotic stress conditions.

Type of Stress	Type of Biostimulant Used	Observed Impact on Plants	Specific Mechanisms Involved	References
Drought	Plant growth-promoting rhizobacteria (PGPR, e.g., *Bacillus* spp., *Pseudomonas* spp.)	Increased root length and enhanced drought resilience	Osmotic adjustment, antioxidant enzyme activity, and change in transcriptome	[151]
Salinity	Mycorrhizal fungi	Improved ionic balance and reduced oxidative damage	Ionic transport and induced systemic tolerance	[152,153]
Heat	VOC-producing bacteria	Enhanced heat tolerance and increased chlorophyll content	Production of volatile organic compounds (VOCs)	[154]
Heavy Metal Toxicity	PGPR (e.g., *Bacillus* spp., *Pseudomonas* spp.)	Reduced metal uptake and decreased oxidative stress	Antioxidant enzyme activity and metal chelation	[151]

## References

[1] Barra Caracciolo A., Terenzi V. (2021). Rhizosphere Microbial Communities and Heavy Metals. Microorganisms.

[2] Santoyo G., Urtis-Flores C.A., Loeza-Lara P.D., Orozco-Mosqueda M.D.C., Glick B.R. (2021). Rhizosphere Colonization Determinants by Plant Growth-Promoting Rhizobacteria (PGPR). Biology.

[3] Ujvári G., Turrini A., Avio L., Agnolucci M. (2021). Possible role of arbuscular mycorrhizal fungi and associated bacteria in the recruitment of endophytic bacterial communities by plant roots. Mycorrhiza.

[4] Akinola S.A., Babalola O.O. (2021). The fungal and archaeal community within plant rhizosphere: A review on their contribution to crop safety. J. Plant Nutr..

[5] Kalleku J.N., Ihsan S., Al-Azzawi T.N.I., Khan M., Hussain A., Chebitok F., Das A.K., Moon Y.S., Mun B.G., Lee I.J. (2024). Halotolerant *Pseudomonas koreensis* S4T10 mitigate salt and drought stress in *Arabidopsis thaliana*. Physiol. Plant..

[6] Ayangbenro A.S., Babalola O.O. (2021). Reclamation of arid and semi-arid soils: The role of plant growth-promoting archaea and bacteria. Curr. Plant Biol..

[7] Ali S., Moon Y.-S., Hamayun M., Khan M.A., Bibi K., Lee I.-J. (2022). Pragmatic role of microbial plant biostimulants in abiotic stress relief in crop plants. J. Plant Interact..

[8] Backer R., Rokem J.S., Ilangumaran G., Lamont J., Praslickova D., Ricci E., Subramanian S., Smith D.L. (2018). Plant Growth-Promoting Rhizobacteria: Context, Mechanisms of Action, and Roadmap to Commercialization of Biostimulants for Sustainable Agriculture. Front. Plant Sci..

[9] Woo S.L., Pepe O. (2018). Microbial consortia: Promising probiotics as plant biostimulants for sustainable agriculture. Front. Plant Sci..

[10] Aamir M., Rai K.K., Zehra A., Dubey M.K., Kumar S., Shukla V., Upadhyay R.S. (2020). Microbial bioformulation-based plant biostimulants: A plausible approach toward next generation of sustainable agriculture. Microbial Endophytes.

[11] Bano A., Waqar A., Khan A., Tariq H. (2022). Phytostimulants in sustainable agriculture. Front. Sustain. Food Syst..

[12] Khalid M.F., Hussain S., Ahmad S., Ejaz S., Zakir I., Ali M.A., Ahmed N., Anjum M.A. (2019). Impacts of abiotic stresses on growth and development of plants. Plant Tolerance to Environmental Stress.

[13] Gull A., Lone A.A., Wani N.U.I. (2019). Biotic and abiotic stresses in plants. Abiotic and Biotic Stress in Plants.

[14] Muhammad M., Waheed A., Wahab A., Majeed M., Nazim M., Liu Y.-H., Li L., Li W.-J. (2024). Soil salinity and drought tolerance: An evaluation of plant growth, productivity, microbial diversity, and amelioration strategies. Plant Stress.

[15] Chaudhry S., Sidhu G.P.S. (2022). Climate change regulated abiotic stress mechanisms in plants: A comprehensive review. Plant Cell Rep..

[16] Drobek M., Frąc M., Cybulska J. (2019). Plant biostimulants: Importance of the quality and yield of horticultural crops and the improvement of plant tolerance to abiotic stress—A review. Agronomy.

[17] Nephali L., Piater L.A., Dubery I.A., Patterson V., Huyser J., Burgess K., Tugizimana F. (2020). Biostimulants for Plant Growth and Mitigation of Abiotic Stresses: A Metabolomics Perspective. Metabolites.

[18] Rai N., Rai S.P., Sarma B.K. (2021). Prospects for abiotic stress tolerance in crops utilizing phyto-and bio-stimulants. Front. Sustain. Food Syst..

[19] Franzoni G., Cocetta G., Prinsi B., Ferrante A., Espen L. (2022). Biostimulants on crops: Their impact under abiotic stress conditions. Horticulturae.

[20] Ansari M., Devi B.M., Sarkar A., Chattopadhyay A., Satnami L., Balu P., Choudhary M., Shahid M.A., Jailani A.A.K. (2023). Microbial exudates as biostimulants: Role in plant growth promotion and stress mitigation. J. Xenobiot..

[21] Montejano-Ramírez V., Ávila-Oviedo J.L., Campos-Mendoza F.J., Valencia-Cantero E. (2024). Microbial Volatile Organic Compounds: Insights into Plant Defense. Plants.

[22] Lephatsi M., Nephali L., Meyer V., Piater L.A., Buthelezi N., Dubery I.A., Opperman H., Brand M., Huyser J., Tugizimana F. (2022). Molecular mechanisms associated with microbial biostimulant-mediated growth enhancement, priming and drought stress tolerance in maize plants. Sci. Rep..

[23] Dar A., Zahir Z.A., Iqbal M., Mehmood A., Javed A., Hussain A., Bushra, Ahmad M. (2021). Efficacy of rhizobacterial exopolysaccharides in improving plant growth, physiology, and soil properties. Environ. Monit. Assess..

[24] Rouphael Y., Colla G. (2020). Biostimulants in agriculture. Front. Plant Sci..

[25] Castiglione A.M., Mannino G., Contartese V., Bertea C.M., Ertani A. (2021). Microbial biostimulants as response to modern agriculture needs: Composition, role and application of these innovative products. Plants.

[26] Sanjuán J., Nápoles M.C., Pérez-Mendoza D., Lorite M.J., Rodríguez-Navarro D.N. (2023). Microbials for agriculture: Why do they call them biostimulants when they mean probiotics?. Microorganisms.

[27] Grammenou A., Petropoulos S.A., Thalassinos G., Rinklebe J., Shaheen S.M., Antoniadis V. (2023). Biostimulants in the soil–plant interface: Agro-environmental implications—A review. Earth Syst. Environ..

[28] Boyno G., Rezaee Danesh Y., Demir S., Teniz N., Mulet J.M., Porcel R. (2023). The Complex Interplay between Arbuscular Mycorrhizal Fungi and Strigolactone: Mechanisms, Sinergies, Applications and Future Directions. Int. J. Mol. Sci..

[29] Dong Y., Zan J., Lin H. (2024). Bioleaching of vanadium from stone coal vanadium ore by *Bacillus mucilaginosus*: Influencing factors and mechanism. Int. J. Miner. Metall. Mater..

[30] Kleinert A., Benedito V., Morcillo R., Dames J., Cornejo-Rivas P., Zuniga-Feest A., Delgado M., Muñoz G. (2018). Morphological and symbiotic root modifications for mineral acquisition from nutrient-poor soils. Root Biology.

[31] Santos T.A.D.C., Menezes G.d.S., Santos J.S., Gois L.d.S., Nascimento S.L.M., Marino R.H. (2018). Microbial Interactions in the development of the biomass of gliricidia. Rev. Caatinga.

[32] Zhang C., van der Heijden M.G.A., Dodds B.K., Nguyen T.B., Spooren J., Valzano-Held A., Cosme M., Berendsen R.L. (2024). A tripartite bacterial-fungal-plant symbiosis in the mycorrhiza-shaped microbiome drives plant growth and mycorrhization. Microbiome.

[33] Soltani J., Zaheri-Shoja M., Hamzei J., Hosseyni-Moghaddam M.S., Pakvaz S., Belbahri L. (2016). Diversity and bioactivity of bacterial endophyte community of Cupressaceae. For. Pathol..

[34] Souza A., Cruz J.C., Sousa N.R., Procópio A.R.L., Silva G.F. (2014). Endophytic bacteria from banana cultivars and their antifungal activity. Genet. Mol. Res..

[35] Kundan R., Pant G., Jadon N., Agrawal P.K. (2015). Plant growth promoting rhizobacteria: Mechanism and current prospective. J. Fertil. Pestic..

[36] Oleńska E., Małek W., Wójcik M., Swiecicka I., Thijs S., Vangronsveld J. (2020). Beneficial features of plant growth-promoting rhizobacteria for improving plant growth and health in challenging conditions: A methodical review. Sci. Total Environ..

[37] Bonaterra A., Badosa E., Daranas N., Francés J., Roselló G., Montesinos E. (2022). Bacteria as biological control agents of plant diseases. Microorganisms.

[38] Wang H., Liu R., You M.P., Barbetti M.J., Chen Y. (2021). Pathogen biocontrol using plant growth-promoting bacteria (PGPR): Role of bacterial diversity. Microorganisms.

[39] Hakim S., Naqqash T., Nawaz M.S., Laraib I., Siddique M.J., Zia R., Mirza M.S., Imran A. (2021). Rhizosphere engineering with plant growth-promoting microorganisms for agriculture and ecological sustainability. Front. Sustain. Food Syst..

[40] Lahlali R., Ezrari S., Radouane N., Kenfaoui J., Esmaeel Q., El Hamss H., Belabess Z., Barka E.A. (2022). Biological Control of Plant Pathogens: A Global Perspective. Microorganisms.

[41] Dimkić I., Janakiev T., Petrović M., Degrassi G., Fira D. (2022). Plant-associated *Bacillus* and *Pseudomonas* antimicrobial activities in plant disease suppression via biological control mechanisms-A review. Physiol. Mol. Plant Pathol..

[42] Munir N., Hanif M., Abideen Z., Sohail M., El-Keblawy A., Radicetti E., Mancinelli R., Haider G. (2022). Mechanisms and strategies of plant microbiome interactions to mitigate abiotic stresses. Agronomy.

[43] Iqbal S., Iqbal M.A., Li C., Iqbal A., Abbas R.N. (2023). Overviewing drought and heat stress amelioration—From plant responses to microbe-mediated mitigation. Sustainability.

[44] Sun W., Shahrajabian M.H., Soleymani A. (2024). The Roles of Plant-Growth-Promoting Rhizobacteria (PGPR)-Based Biostimulants for Agricultural Production Systems. Plants.

[45] Bhantana P., Rana M.S., Sun X.-c., Moussa M.G., Saleem M.H., Syaifudin M., Shah A., Poudel A., Pun A.B., Bhat M.A. (2021). Arbuscular mycorrhizal fungi and its major role in plant growth, zinc nutrition, phosphorous regulation and phytoremediation. Symbiosis.

[46] Khaliq A., Perveen S., Alamer K.H., Zia Ul Haq M., Rafique Z., Alsudays I.M., Althobaiti A.T., Saleh M.A., Hussain S., Attia H. (2022). Arbuscular mycorrhizal fungi symbiosis to enhance plant–soil interaction. Sustainability.

[47] Hu Y., Pandey A.K., Wu X., Fang P., Xu P. (2022). The role of arbuscular mycorrhiza fungi in drought tolerance in legume crops: A review. Legume Res.—Int. J..

[48] Devi S.H., Bhupenchandra I., Sinyorita S., Chongtham S., Devi E.L. (2021). Mycorrhizal fungi and sustainable agriculture. Nitrogen in Agriculture—Physiological, Agricultural and Ecological Aspects.

[49] Ortas I., Rafique M., Çekiç F. (2021). Do Mycorrhizal Fungi enable plants to cope with abiotic stresses by overcoming the detrimental effects of salinity and improving drought tolerance?. Symbiotic Soil Microorganisms: Biology and Applications.

[50] Wahab A., Muhammad M., Munir A., Abdi G., Zaman W., Ayaz A., Khizar C., Reddy S.P.P. (2023). Role of Arbuscular Mycorrhizal Fungi in Regulating Growth, Enhancing Productivity, and Potentially Influencing Ecosystems under Abiotic and Biotic Stresses. Plants.

[51] Wahab A., Abdi G., Saleem M.H., Ali B., Ullah S., Shah W., Mumtaz S., Yasin G., Muresan C.C., Marc R.A. (2022). Plants’ physio-biochemical and phyto-hormonal responses to alleviate the adverse effects of drought stress: A comprehensive review. Plants.

[52] Monib A.W., Niazi P., Barai S.M., Sawicka B., Baseer A.Q., Nikpay A., Fahmawi S.M.S., Singh D., Alikhail M., Thea B. (2024). Nitrogen Cycling Dynamics: Investigating Volatilization and its Interplay with N2 Fixation. J. Res. Appl. Sci. Biotechnol..

[53] Achari G.A. (2020). The role of plant-associated bacteria. Global Implications of the Nitrogen Cycle.

[54] Jehani M., Singh S., Archana T., Kumar D., Kumar G. (2023). Azospirillum—A free-living nitrogen-fixing bacterium. Rhizobiome.

[55] Sun W., Shahrajabian M.H., Cheng Q. (2021). Nitrogen fixation and diazotrophs—A review. Rom. Biotechnol. Lett..

[56] Vats S., Srivastava P., Saxena S., Mudgil B., Kumar N. (2021). Beneficial Effects of Nitrogen-Fixing Bacteria for Agriculture of the Future. Soil Nitrogen Ecology.

[57] Mishra P., Mishra J., Arora N.K. (2021). Plant growth promoting bacteria for combating salinity stress in plants–Recent developments and prospects: A review. Microbiol. Res..

[58] Compant S., Cambon M.C., Vacher C., Mitter B., Samad A., Sessitsch A. (2021). The plant endosphere world–bacterial life within plants. Environ. Microbiol..

[59] Khan S.S., Verma V., Rasool S. (2020). Diversity and the role of endophytic bacteria: A review. Bot. Serbica.

[60] Tripathi A., Pandey P., Tripathi S.N., Kalra A. (2022). Perspectives and potential applications of endophytic microorganisms in cultivation of medicinal and aromatic plants. Front. Plant Sci..

[61] Singh M., Srivastava M., Kumar A., Singh A., Pandey K. (2020). Endophytic bacteria in plant disease management. Microbial Endophytes.

[62] Kumar V., Nautiyal C.S. (2022). Plant Abiotic and Biotic Stress Alleviation: From an Endophytic Microbial Perspective. Curr. Microbiol..

[63] Phurailatpam L., Mishra S. (2020). Role of plant endophytes in conferring abiotic stress tolerance. Plant Ecophysiology and Adaptation Under Climate Change: Mechanisms and Perspectives II: Mechanisms of Adaptation and Stress Amelioration.

[64] Salvi P., Mahawar H., Agarrwal R., Kajal, Gautam V., Deshmukh R. (2022). Advancement in the molecular perspective of plant-endophytic interaction to mitigate drought stress in plants. Front. Microbiol..

[65] Verma S.K., Sahu P.K., Kumar K., Pal G., Gond S.K., Kharwar R.N., White J.F. (2021). Endophyte roles in nutrient acquisition, root system architecture development and oxidative stress tolerance. J. Appl. Microbiol..

[66] Kaushal P., Ali N., Saini S., Pati P.K., Pati A.M. (2023). Physiological and molecular insight of microbial biostimulants for sustainable agriculture. Front. Plant Sci..

[67] Lynch J.P. (2022). Edaphic stress interactions: Important yet poorly understood drivers of plant production in future climates. Field Crops Res..

[68] Wong W.S., Zhong H.T., Cross A.T., Yong J.W.H. (2020). Plant biostimulants in vermicomposts: Characteristics and plausible mechanisms. The Chemical Biology of Plant Biostimulants.

[69] Oyedele A.O., Ezaka E., Taiwo L.B. (2024). Microbial biosynthesis of the classical phytohormones by plant growth-promoting microorganisms in plants. Microbial Biostimulants for Plant Growth and Abiotic Stress Amelioration.

[70] Druege U. (2020). Overcoming Physiological Bottlenecks of Leaf Vitality and Root Development in Cuttings: A Systemic Perspective. Front. Plant Sci..

[71] Jalil S.U., Ansari S.A., Ansari M.I. (2022). Role of environment stress leaf senescence and crop productivity. Augmenting Crop Productivity in Stress Environment.

[72] Ranjan A., Rajput V.D., Prazdnova E.V., Gurnani M., Sharma S., Bhardwaj P., Shende S.S., Mandzhieva S.S., Sushkova S., Minkina T. (2024). Augmenting abiotic stress tolerance and root architecture: The function of phytohormone-producing PGPR and their interaction with nanoparticles. S. Afr. J. Bot..

[73] Frederix M. (2010). A Novel Mechanism of Coupling Quorum Sensing Systems in *Rhizobium leguminosarum* bv. *viciae* 3841. Doctoral Dissertation.

[74] Shekhawat K., Fröhlich K., García-Ramírez G.X., Trapp M.A., Hirt H. (2022). Ethylene: A master regulator of plant–microbe interactions under abiotic stresses. Cells.

[75] Iqbal B., Li G., Alabbosh K.F., Hussain H., Khan I., Tariq M., Javed Q., Naeem M., Ahmad N. (2023). Advancing environmental sustainability through microbial reprogramming in growth improvement, stress alleviation, and phytoremediation. Plant Stress.

[76] Hasanuzzaman M., Parvin K., Bardhan K., Nahar K., Anee T.I., Masud A.A.C., Fotopoulos V. (2021). Biostimulants for the Regulation of Reactive Oxygen Species Metabolism in Plants under Abiotic Stress. Cells.

[77] Melo-Sabogal D.V., Contreras-Medina L.M. (2024). Elicitors and Biostimulants to Mitigate Water Stress in Vegetables. Horticulturae.

[78] Martínez-Lorente S.E., Martí-Guillén J.M., Pedreño M.Á., Almagro L., Sabater-Jara A.B. (2024). Higher Plant-Derived Biostimulants: Mechanisms of Action and Their Role in Mitigating Plant Abiotic Stress. Antioxidants.

[79] Bitterlich M., Mercy L., Arato M., Franken P. (2020). Arbuscular mycorrhizal fungi as biostimulants for sustainable crop production. Biostimulants for Sustainable Crop Production.

[80] Rouphael Y., Colla G. (2020). Toward a sustainable agriculture through plant biostimulants: From experimental data to practical applications. Agronomy.

[81] Olowe O.M., Akanmu A.O., Asemoloye M.D. (2020). Exploration of microbial stimulants for induction of systemic resistance in plant disease management. Ann. Appl. Biol..

[82] Yu Y., Gui Y., Li Z., Jiang C., Guo J., Niu D. (2022). Induced Systemic Resistance for Improving Plant Immunity by Beneficial Microbes. Plants.

[83] Mrid R.B., Benmrid B., Hafsa J., Boukcim H., Sobeh M., Yasri A. (2021). Secondary metabolites as biostimulant and bioprotectant agents: A review. Sci. Total Environ..

[84] Kumari M., Swarupa P., Kesari K.K., Kumar A. (2022). Microbial inoculants as plant biostimulants: A review on risk status. Life.

[85] Mostafa A.A., El-Rahman S.N.A., Shehata S., Abdallah N.A., Omar H.S. (2021). Assessing the effects of a novel biostimulant to enhance leafminer resistance and plant growth on common bean. Sci. Rep..

[86] Singh A., Singh S., Kurella A., Verma A., Mahatama M., Venkatesh I. (2022). Plant bio-stimulants, their functions and use in enhancing stress tolerance in oilseeds. New and Future Developments in Microbial Biotechnology and Bioengineering.

[87] Ali S., Tyagi A., Park S., Mir R.A., Mushtaq M., Bhat B., Mahmoudi H., Bae H. (2022). Deciphering the plant microbiome to improve drought tolerance: Mechanisms and perspectives. Environ. Exp. Bot..

[88] Islam M.T., Gan H.M., Ziemann M., Hussain H.I., Arioli T., Cahill D. (2020). Phaeophyceaean (brown algal) extracts activate plant defense systems in *Arabidopsis thaliana* challenged with *Phytophthora cinnamomi*. Front. Plant Sci..

[89] Zehra A., Raytekar N.A., Meena M., Swapnil P. (2021). Efficiency of microbial bio-agents as elicitors in plant defense mechanism under biotic stress: A review. Curr. Res. Microb. Sci..

[90] Maithani D., Singh H., Sharma A. (2021). Stress alleviation in plants using SAR and ISR: Current views on stress signaling network. Microbes and Signaling Biomolecules Against Plant Stress: Strategies of Plant-Microbe Relationships for Better Survival.

[91] Yadav S., Modi P., Dave A., Vijapura A., Patel D., Patel M. (2020). Effect of abiotic stress on crops. Sustain. Crop Prod..

[92] Das R., Biswas S. (2022). Influence of abiotic stresses on seed production and quality. Seed Biology Updates.

[93] Bharti R., Sharma D., Kunjam S. (2024). Role of abiotic and biotic stress management practices in extreme environment in monitoring and improving physiological properties of plant. Modern Techniques To Sustainable Agriculture.

[94] Rathod A., Verma N.S. (2023). Impact of Abiotic Stress on Agronomical Crops. Frontiers of Agronomy.

[95] Sarkar S., Saha S., Ghosh S., Paul S.K., Dey S., Moulick D., Santra S.C., Brahmachari K. (2023). Abiotic stress sensitivity and adaptation in field crops. Climate-Resilient Agriculture, Vol 2: Agro-Biotechnological Advancement for Crop Production.

[96] Ashapkin V.V., Kutueva L.I., Aleksandrushkina N.I., Vanyushin B.F. (2020). Epigenetic Mechanisms of Plant Adaptation to Biotic and Abiotic Stresses. Int. J. Mol. Sci..

[97] Seleiman M.F., Al-Suhaibani N., Ali N., Akmal M., Alotaibi M., Refay Y., Dindaroglu T., Abdul-Wajid H.H., Battaglia M.L. (2021). Drought Stress Impacts on Plants and Different Approaches to Alleviate Its Adverse Effects. Plants.

[98] Oguz M.C., Aycan M., Oguz E., Poyraz I., Yildiz M. (2022). Drought stress tolerance in plants: Interplay of molecular, biochemical and physiological responses in important development stages. Physiologia.

[99] Shoaib M., Banerjee B.P., Hayden M., Kant S. (2022). Roots’ drought adaptive traits in crop improvement. Plants.

[100] Halpern M., Bar-Tal A., Ofek M., Minz D., Muller T., Yermiyahu U. (2015). The use of biostimulants for enhancing nutrient uptake. Adv. Agron..

[101] Yadav S., Irfan M., Ahmad A., Hayat S. (2011). Causes of salinity and plant manifestations to salt stress: A review. J. Environ. Biol..

[102] Balasubramaniam T., Shen G., Esmaeili N., Zhang H. (2023). Plants’ response mechanisms to salinity stress. Plants.

[103] Yildiz M., Poyraz İ., Çavdar A., Özgen Y., Beyaz R. (2020). Plant Responses to Salt Stress.

[104] Gul S.L., Moon Y.S., Hamayun M., Khan S.A., Iqbal A., Khan M.A., Hussain A., Shafique M., Kim Y.H., Ali S. (2022). Porostereum spadiceum-AGH786 Regulates the Growth and Metabolites Production in Triticum aestivum L. Under Salt Stress. Curr. Microbiol..

[105] Khan A., Ali S., Khan M., Hamayun M., Moon Y.-S. (2022). Parthenium hysterophorus’s endophytes: The second layer of defense against biotic and abiotic stresses. Microorganisms.

[106] Musa M., Jan F.G., Hamayun M., Jan G., Khan S.A., Rehman G., Ali S., Lee I.-J. (2023). An endophytic fungal isolate Paecilomyces lilacinus produces bioactive secondary metabolites and promotes growth of Solanum lycopersicum under heavy metal stress. Agronomy.

[107] Bhattacharya A. (2022). Physiological Processes in Plants Under Low Temperature Stress.

[108] Soualiou S., Duan F., Li X., Zhou W. (2022). Crop production under cold stress: An understanding of plant responses, acclimation processes, and management strategies. Plant Physiol. Biochem..

[109] Ul Hassan M., Rasool T., Iqbal C., Arshad A., Abrar M., Abrar M.M., Habib-ur-Rahman M., Noor M.A., Sher A., Fahad S. (2021). Linking plants functioning to adaptive responses under heat stress conditions: A mechanistic review. J. Plant Growth Regul..

[110] Aslam M.A., Ahmed M., Hassan F.-U., Afzal O., Mehmood M.Z., Qadir G., Asif M., Komal S., Hussain T. (2022). Impact of temperature fluctuations on plant morphological and physiological traits. Building Climate Resilience in Agriculture: Theory, Practice and Future Perspective.

[111] Rasheed A., Hassan M.U., Fahad S., Aamer M., Batool M., Ilyas M., Shang F., Wu Z., Li H. (2021). Heavy metals stress and plants defense responses. Sustainable Soil and Land Management and Climate Change.

[112] Aziz L., Hamayun M., Rauf M., Iqbal A., Husssin A., Khan S.A., Shafique M., Arif M., Ahmad A., Rehman G. (2022). Aspergillus violaceofuscus alleviates cadmium and chromium stress in Okra through biochemical modulation. PLoS ONE.

[113] Muhammad L., Khan A., Zhou Y., He M., Alrefaei A.F., Khan M., Ali S. (2023). Physiological and ultrastructural changes in Dendranthema morifolium cultivars exposed to different cadmium stress conditions. Agriculture.

[114] Kocaman A. (2023). Combined interactions of amino acids and organic acids in heavy metal binding in plants. Plant Signal. Behav..

[115] Raza A., Hussain S., Javed R., Hafeez M.B., Hasanuzzaman M. (2021). Antioxidant defense systems and remediation of metal toxicity in plants. Approaches to the Remediation of Inorganic Pollutants.

[116] Kosakivska I.V., Babenko L.M., Romanenko K.O., Korotka I.Y., Potters G. (2021). Molecular mechanisms of plant adaptive responses to heavy metals stress. Cell Biol. Int..

[117] Yoon Y., Seo D.H., Shin H., Kim H.J., Kim C.M., Jang G. (2020). The role of stress-responsive transcription factors in modulating abiotic stress tolerance in plants. Agronomy.

[118] Sharma M., Kumar P., Verma V., Sharma R., Bhargava B., Irfan M. (2022). Understanding plant stress memory response for abiotic stress resilience: Molecular insights and prospects. Plant Physiol. Biochem..

[119] Robredo A., Pérez-López U., Lacuesta M., Mena-Petite A., Muñoz-Rueda A. (2010). Influence of water stress on photosynthetic characteristics in barley plants under ambient and elevated CO2 concentrations. Biol. Plant..

[120] Flexas J., Bota J., Galmés J., Medrano H., Ribas-Carbó M. (2006). Keeping a positive carbon balance under adverse conditions: Responses of photosynthesis and respiration to water stress. Physiol. Plant..

[121] Silva E.N., Ribeiro R.V., Ferreira-Silva S.L., Viégas R.A., Silveira J.A.G. (2010). Comparative effects of salinity and water stress on photosynthesis, water relations and growth of Jatropha curcas plants. J. Arid Environ..

[122] Zouaoui R., Ammari Y., Abassi M., Ahmed H.B., Smaoui A., Hilali K. (2019). Physiological and biochemical responses of rhus tripartita (Ucria) grande under water stress. Pak. J. Bot..

[123] Nouri M.-Z., Moumeni A., Komatsu S. (2015). Abiotic Stresses: Insight into Gene Regulation and Protein Expression in Photosynthetic Pathways of Plants. Int. J. Mol. Sci..

[124] Chauhan J., Prathibha M.D., Singh P., Choyal P., Mishra U.N., Saha D., Kumar R., Anuragi H., Pandey S., Bose B. (2023). Plant photosynthesis under abiotic stresses: Damages, adaptive, and signaling mechanisms. Plant Stress.

[125] Duque A.S., de Almeida M., da Silva A.B., da Silva J.M., Farinha A.P., Santos D., Fevereiro P., de Sousa Arau S., Kourosh V., Charles L. (2013). Abiotic Stress Responses in Plants: Unraveling the Complexity of Genes and Networks to Survive. Abiotic Stress.

[126] Kumar S. (2020). Abiotic stresses and their effects on plant growth, yield and nutritional quality of agricultural produce. Int. J. Food Sci. Agric..

[127] Vega J.M., Garbayo I., Domínguez M.J., Vigara J. (2006). Effect of abiotic stress on photosynthesis and respiration in Chlamydomonas reinhardtii. Enzym. Microb. Technol..

[128] Pawlowicz I., Masajada K. (2019). Aquaporins as a link between water relations and photosynthetic pathway in abiotic stress tolerance in plants. Gene.

[129] Khan N., Ali S., Shahid M.A., Mustafa A., Sayyed R., Curá J.A. (2021). Insights into the interactions among roots, rhizosphere, and rhizobacteria for improving plant growth and tolerance to abiotic stresses: A review. Cells.

[130] Sharma I., Kashyap S., Agarwala N. (2023). Biotic stress-induced changes in root exudation confer plant stress tolerance by altering rhizospheric microbial community. Front. Plant Sci..

[131] Olanrewaju O.S., Babalola O.O. (2022). The rhizosphere microbial complex in plant health: A review of interaction dynamics. J. Integr. Agric..

[132] Marín O., González B., Poupin M.J. (2021). From microbial dynamics to functionality in the rhizosphere: A systematic review of the opportunities with synthetic microbial communities. Front. Plant Sci..

[133] Jain A., Chakraborty J., Das S. (2020). Underlying mechanism of plant–microbe crosstalk in shaping microbial ecology of the rhizosphere. Acta Physiol. Plant..

[134] Lyu D., Smith D.L. (2022). The root signals in rhizospheric inter-organismal communications. Front. Plant Sci..

[135] Ma W., Tang S., Dengzeng Z., Zhang D., Zhang T., Ma X. (2022). Root exudates contribute to belowground ecosystem hotspots: A review. Front. Microbiol..

[136] Afridi M.S., Kumar A., Javed M.A., Dubey A., de Medeiros F.H.V., Santoyo G. (2024). Harnessing root exudates for plant microbiome engineering and stress resistance in plants. Microbiol. Res..

[137] Sun H., Jiang S., Jiang C., Wu C., Gao M., Wang Q. (2021). A review of root exudates and rhizosphere microbiome for crop production. Environ. Sci. Pollut. Res. Int..

[138] DeFalco T.A., Zipfel C. (2021). Molecular mechanisms of early plant pattern-triggered immune signaling. Mol. Cell.

[139] Chiquito-Contreras C.J., Meza-Menchaca T., Guzmán-López O., Vásquez E.C., Ricaño-Rodríguez J. (2024). Molecular insights into plant–microbe interactions: A comprehensive review of key mechanisms. Front. Biosci.-Elite.

[140] Wu S., Liu J., Liu C., Yang A., Qiao J. (2020). Quorum sensing for population-level control of bacteria and potential therapeutic applications. Cell. Mol. Life Sci..

[141] Moreno-Gámez S., Hochberg M.E., Van Doorn G. (2023). Quorum sensing as a mechanism to harness the wisdom of the crowds. Nat. Commun..

[142] Nag M., Lahiri D., Mukherjee D., Banerjee R., Garai S., Sarkar T., Ghosh S., Dey A., Ghosh S., Pattnaik S. (2021). Functionalized Chitosan Nanomaterials: A Jammer for Quorum Sensing. Polymers.

[143] Fincheira P., Quiroz A., Tortella G., Diez M.C., Rubilar O. (2021). Current advances in plant-microbe communication via volatile organic compounds as an innovative strategy to improve plant growth. Microbiol. Res..

[144] Contreras-Cornejo H.A., Ramírez-Ordorica A., Álvarez-Navarrete M., Macías-Rodríguez L. (2022). The role of Phytohormones in cross-communication between plants and Rhizo-microbes. Microbial Cross-Talk in the Rhizosphere.

[145] Poveda J., González-Andrés F. (2021). *Bacillus* as a source of phytohormones for use in agriculture. Appl. Microbiol. Biotechnol..

[146] Poveda J. (2021). Beneficial effects of microbial volatile organic compounds (MVOCs) in plants. Appl. Soil Ecol..

[147] Srikamwang C., Onsa N.E., Sunanta P., Sangta J., Chanway C.P., Thanakkasaranee S., Sommano S.R. (2023). Role of Microbial Volatile Organic Compounds in Promoting Plant Growth and Disease Resistance in Horticultural Production. Plant Signal. Behav..

[148] Sajna K.V., Sharma S., Nadda A.K. (2021). Microbial exopolysaccharides: An introduction. Microbial Exopolysaccharides as Novel and Significant Biomaterials.

[149] Prasad S., Purohit S.R. (2023). Microbial exopolysaccharide: Sources, stress conditions, properties and application in food and environment: A comprehensive review. Int. J. Biol. Macromol..

[150] Mall M., Kumar R., Akhtar M.Q. (2021). Horticultural crops and abiotic stress challenges. Stress Tolerance in Horticultural Crops.

[151] Dimkpa C., Weinand T., Asch F. (2009). Plant-rhizobacteria interactions alleviate abiotic stress conditions. Plant Cell Environ..

[152] Maheshwari H.S., Bharti A., Agnihotri R., Dukare A., Prabina B.J., Gangola S., Sharma M.P. (2021). Combating the Abiotic Stress Through Phytomicrobiome Studies. Phytomicrobiome Interactions and Sustainable Agriculture.

[153] Teklić T., Parađiković N., Špoljarević M., Zeljković S., Lončarić Z., Lisjak M. (2020). Linking abiotic stress, plant metabolites, biostimulants and functional food. Ann. Appl. Biol..

[154] Andrade-Linares D.R., Lehmann A., Rillig M.C. (2016). Microbial stress priming: A meta-analysis. Environ. Microbiol..

[155] Ozturk M., Turkyilmaz Unal B., Garcia-Caparros P., Khursheed A., Gul A., Hasanuzzaman M. (2021). Osmoregulation and its actions during the drought stress in plants. Physiol. Plant..

[156] Kashyap S., Biswal B., Bhakuni K., Ali G., Bhattacharjee S., Yadav M.R., Kumar R. (2024). Amelioration of abiotic stresses in forage crop production using microbial stimulants: An overview. Microbial Biostimulants for Plant Growth and Abiotic Stress Amelioration.

[157] Kempf B., Bremer E. (1998). Uptake and synthesis of compatible solutes as microbial stress responses to high-osmolality environments. Arch. Microbiol..

[158] Barnawal D., Singh R., Singh R.P. (2019). Role of plant growth promoting rhizobacteria in drought tolerance: Regulating growth hormones and osmolytes. PGPR Amelioration in Sustainable Agriculture.

[159] Gill S.S., Tuteja N. (2010). Reactive oxygen species and antioxidant machinery in abiotic stress tolerance in crop plants. Plant Physiol. Biochem..

[160] Thiruvengadam R., Venkidasamy B., Easwaran M., Chi H.Y., Thiruvengadam M., Kim S.H. (2024). Dynamic interplay of reactive oxygen and nitrogen species (ROS and RNS) in plant resilience: Unveiling the signaling pathways and metabolic responses to biotic and abiotic stresses. Plant Cell Rep..

[161] Kesawat M.S., Satheesh N., Kherawat B.S., Kumar A., Kim H.-U., Chung S.-M., Kumar M. (2023). Regulation of reactive oxygen species during salt stress in plants and their crosstalk with other signaling molecules—Current perspectives and future directions. Plants.

[162] Etesami H., Jeong B.R., Glick B.R. (2023). Potential use of Bacillus spp. as an effective biostimulant against abiotic stresses in crops—A review. Curr. Res. Biotechnol..

[163] Shahrajabian M.H., Petropoulos S.A., Sun W. (2023). Survey of the influences of microbial biostimulants on horticultural crops: Case studies and successful paradigms. Horticulturae.

[164] Kumar M., Gupta A., Vandana P., Tiwari L.D., Patel M.K., Siddique K.H. (2024). Recent advances of plant growth-promoting rhizobacteria (PGPR)-mediated drought and waterlogging stress tolerance in plants for sustainable agriculture. Microbial Biostimulants for Plant Growth and Abiotic Stress Amelioration.

[165] Eswaran S.U.D., Sundaram L., Perveen K., Bukhari N.A., Sayyed R.Z. (2024). Osmolyte-producing microbial biostimulants regulate the growth of Arachis hypogaea L. under drought stress. BMC Microbiol..

[166] Hualpa-Ramirez E., Carrasco-Lozano E.C., Madrid-Espinoza J., Tejos R., Ruiz-Lara S., Stange C., Norambuena L. (2024). Stress salinity in plants: New strategies to cope with in the foreseeable scenario. Plant Physiol. Biochem..

[167] Sharma K., Saryam M., Kumar M., Sharma P.K., Prasad S.R. (2024). Salt-Adapted Plant Growth Promoting Rhizo-bacteria (SA-PGPR): A systemic approach towards mitigation of salinity. Ecol. Environ. Conserv..

[168] Kohlstedt M., Sappa P.K., Meyer H., Maaß S., Zaprasis A., Hoffmann T., Becker J., Steil L., Hecker M., van Dijl J.M. (2014). Adaptation of B acillus subtilis carbon core metabolism to simultaneous nutrient limitation and osmotic challenge: A multi-omics perspective. Environ. Microbiol..

[169] Mishra A.K., Das R., George Kerry R., Biswal B., Sinha T., Sharma S., Arora P., Kumar M. (2023). Promising management strategies to improve crop sustainability and to amend soil salinity. Front. Environ. Sci..

[170] Buragohain K., Tamuly D., Sonowal S., Nath R. (2024). Impact of Drought Stress on Plant Growth and Its Management Using Plant Growth Promoting Rhizobacteria. Indian J. Microbiol..

[171] Huang J.L., Li Z.Y., Mao J.Y., Chen Z.M., Liu H.L., Liang G.Y., Zhang D.B., Wen P.J., Mo Z.Y., Jiang Y.M. (2024). Contamination and health risks brought by arsenic, lead and cadmium in a water-soil-plant system nearby a non-ferrous metal mining area. Ecotoxicol. Environ. Saf..

[172] Gupta R., Khan F., Alqahtani F.M., Hashem M., Ahmad F. (2024). Plant Growth-Promoting Rhizobacteria (PGPR) Assisted Bioremediation of Heavy Metal Toxicity. Appl. Biochem. Biotechnol..

[173] Garg S., Nain P., Kumar A., Joshi S., Punetha H., Sharma P.K., Siddiqui S., Alshaharni M.O., Algopishi U.B., Mittal A. (2024). Next generation plant biostimulants & genome sequencing strategies for sustainable agriculture development. Front. Microbiol..

[174] Esposito A., Colantuono C., Ruggieri V., Chiusano M.L. (2016). Bioinformatics for agriculture in the next-generation sequencing era. Chem. Biol. Technol. Agric..

[175] Mandal S., Anand U., Lopez-Bucio J., Radha, Kumar M., Lal M.K., Tiwari R.K., Dey A. (2023). Biostimulants and environmental stress mitigation in crops: A novel and emerging approach for agricultural sustainability under climate change. Environ. Res..

[176] Bertoldo G. (2023). Omics Approach to Dissect Complex Morpho-Physiological and Molecular Responses to Nutritional and Biostimulant Stimuli in Plants. Ph.D. Thesis.

[177] Adeleke B.S., Muller D., Babalola O.O. (2023). A metagenomic lens into endosphere microbial communities, promises, and discoveries. Lett. Appl. Microbiol..

[178] Rajguru B., Shri M., Bhatt V.D. (2024). Exploring microbial diversity in the rhizosphere: A comprehensive review of metagenomic approaches and their applications. 3 Biotech.

[179] Iosa I., Agrimonti C., Marmiroli N. (2024). Real-Time PCR (qtPCR) to Discover the Fate of Plant Growth-Promoting Rhizobacteria (PGPR) in Agricultural Soils. Microorganisms.

[180] Ujvári G., Capo L., Grassi A., Cristani C., Pagliarani I., Turrini A., Blandino M., Giovannetti M., Agnolucci M. (2023). Agronomic strategies to enhance the early vigor and yield of maize. Part I: The role of seed applied biostimulant, hybrid and starter fertilization on rhizosphere bacteria profile and diversity. Front. Plant Sci..

[181] Wójcik M., Koper P., Żebracki K., Marczak M., Mazur A. (2023). Genomic and Metabolic Characterization of Plant Growth-Promoting Rhizobacteria Isolated from Nodules of Clovers Grown in Non-Farmed Soil. Int. J. Mol. Sci..

[182] Patel A., Sahu K.P., Mehta S., Javed M., Balamurugan A., Ashajyothi M., Sheoran N., Ganesan P., Kundu A., Gopalakrishnan S. (2023). New Insights on Endophytic Microbacterium-Assisted Blast Disease Suppression and Growth Promotion in Rice: Revelation by Polyphasic Functional Characterization and Transcriptomics. Microorganisms.

[183] Satrio R.D., Fendiyanto M.H., Miftahudin M. (2024). Tools and Techniques Used at Global Scale Through Genomics, Transcriptomics, Proteomics, and Metabolomics to Investigate Plant Stress Responses at the Molecular Level. Molecular Dynamics of Plant Stress and Its Management.

[184] Rizvi M.Z., Abid M., Pandey S., Abid Ali Khan M. (2024). Role of Transcriptomics in Elucidating Mechanism of Abiotic Stress Tolerance in Plants. Microbial Biotechnology for Sustainable Agriculture Volume 2.

[185] Husen A., Ahmad A. (2023). Genomics, Transcriptomics, Proteomics and Metabolomics of Crop Plants.

[186] Choi H.W., Klessig D.F. (2016). DAMPs, MAMPs, and NAMPs in plant innate immunity. BMC Plant Biol..

[187] Tu M., Du C., Yu B., Wang G., Deng Y., Wang Y., Chen M., Chang J., Yang G., He G. (2023). Current advances in the molecular regulation of abiotic stress tolerance in sorghum via transcriptomic, proteomic, and metabolomic approaches. Front. Plant Sci..

[188] Aslam N., Li Q., Bashir S., Yuan L., Qiao L., Li W. (2024). Integrated Review of Transcriptomic and Proteomic Studies to Understand Molecular Mechanisms of Rice’s Response to Environmental Stresses. Biology.

[189] Ansori A.N., Antonius Y., Susilo R.J., Hayaza S., Kharisma V.D., Parikesit A.A., Zainul R., Jakhmola V., Saklani T., Rebezov M. (2023). Application of CRISPR-Cas9 genome editing technology in various fields: A review. Narra J.

[190] Li J., Wu S., Zhang K., Sun X., Lin W., Wang C., Lin S. (2024). Clustered regularly interspaced short palindromic repeat/crispr-associated protein and its utility all at sea: Status, challenges, and prospects. Microorganisms.

[191] Irfan M., Majeed H., Iftikhar T., Ravi P.K. (2024). A review on molecular scissoring with CRISPR/Cas9 genome editing technology. Toxicol. Res..

[192] Lee T.M., Lin J.Y., Tsai T.H., Yang R.Y., Ng I.S. (2023). Clustered regularly interspaced short palindromic repeats (CRISPR) technology and genetic engineering strategies for microalgae towards carbon neutrality: A critical review. Bioresour. Technol..

[193] Maduelosi B.I. (2024). The Impact of Clustered Regularly Interspaced Short Palindromic Repeats (CRISPR) in Pharmacy. Master’s Thesis.

[194] Thakur N., Nigam M., Mann N.A., Gupta S., Hussain C.M., Shukla S.K., Shah A.A., Casini R., Elansary H.O., Khan S.A. (2023). Host-mediated gene engineering and microbiome-based technology optimization for sustainable agriculture and environment. Funct. Integr. Genom..

[195] Khan A., Pan X., Najeeb U., Tan D.K.Y., Fahad S., Zahoor R., Luo H. (2018). Coping with drought: Stress and adaptive mechanisms, and management through cultural and molecular alternatives in cotton as vital constituents for plant stress resilience and fitness. Biol. Res..

[196] Bhuyan S.J., Kumar M., Ramrao Devde P., Rai A.C., Mishra A.K., Singh P.K., Siddique K.H.M. (2023). Progress in gene editing tools, implications and success in plants: A review. Front. Genome Ed..

[197] Bose J.C., Sarwan J., Narang J., Mittal K., Sharma H. (2023). Futuristic Approaches in Biofertilizer Industry Through Metabolomics, Proteomes, and Gene Editing. Metabolomics, Proteomes and Gene Editing Approaches in Biofertilizer Industry.

[198] Srikanth P., Sivakumar D., Sharma A., Kaushik N. (2024). Recent developments in omics techniques for improving plant abiotic stress using microbes. Int. J. Environ. Sci. Technol..

[199] Kaya C. (2024). Microbial modulation of hormone signaling, proteomic dynamics, and metabolomics in plant drought adaptation. Food Energy Secur..

[200] Afridi M.S., Ali S., Salam A., César Terra W., Hafeez A., Sumaira, Ali B., AlTami M.S., Ameen F., Ercisli S. (2022). Plant microbiome engineering: Hopes or hypes. Biology.

[201] Malviya D., Ilyas T., Chaurasia R., Singh U.B., Shahid M., Vishwakarma S.K., Shafi Z., Yadav B., Sharma S.K., Singh H.V. (2022). Engineering the plant microbiome for biotic stress tolerance: Biotechnological advances. Rhizosphere Microbes: Biotic Stress Management.

[202] Johnson R., Joel J.M., Puthur J.T. (2024). Biostimulants: The futuristic sustainable approach for alleviating crop productivity and abiotic stress tolerance. J. Plant Growth Regul..

[203] Upadhyay S.K., Rajput V.D., Kumari A., Espinosa-Saiz D., Menendez E., Minkina T., Dwivedi P., Mandzhieva S. (2023). Plant growth-promoting rhizobacteria: A potential bio-asset for restoration of degraded soil and crop productivity with sustainable emerging techniques. Environ. Geochem. Health.

[204] Kashyap S., Barman R., Nath M., Agarwala N. (2023). Microbial symbionts for alleviation of heavy metal toxicity in crop plants. Biostimulants in Alleviation of Metal Toxicity in Plants.

[205] Li J. (2024). Biostimulant Discovery from Agricultural Biomass. Doctoral Dissertation.

[206] Sun W., Shahrajabian M.H. (2023). The application of arbuscular mycorrhizal fungi as microbial biostimulant, sustainable approaches in modern agriculture. Plants.

[207] Iannone L.J., Novas M.V., Mc Cargo P.D., Ueno A.C., Gundel P.E. (2021). Diversity, Ecology, and Applications of Epichloë Fungal Endophytes of Grasses in South America. Neotropical Endophytic Fungi: Diversity, Ecology, and Biotechnological Applications.

[208] Perez-Lamarque B., Petrolli R., Strullu-Derrien C., Strasberg D., Morlon H., Selosse M., Martos F. (2021). Fungal sharing, specialization, and structural distinctiveness in the plant root microbiomes of distantly related plant lineages. Caractérisation Modélisation L’évolution Interact. Hôtes-Microbiotes.

[209] Suárez Díaz J. (2020). Methodological Strategies in Contemporary Symbiosis Research and Their Historical Roots: From Mechanistic to Non-Mechanistic Modes of Explanation. Doctoral Dissertation.

[210] Santini G., Biondi N., Rodolfi L., Tredici M.R. (2021). Plant biostimulants from cyanobacteria: An emerging strategy to improve yields and sustainability in agriculture. Plants.

[211] Wang S., Zhan Y., Jiang X., Lai Y. (2024). Engineering Microbial Consortia as Living Materials: Advances and Prospectives. ACS Synth. Biol..

[212] Pejcz E. (2024). Biotechnological Approach of Technological Advancements for Sustainable Probiotic Bread Production. Sustainability.

[213] Somna G., Challabathula D., Bakka K. (2023). Nanobiofertilizers: Applications, Crop Productivity, and Sustainable Agriculture. Nanofertilizers for Sustainable Agroecosystems: Recent Advances and Future Trends.

[214] Koul O. (2023). Development and Commercialization of Biopesticides: Costs and Benefits.

[215] Sangiorgio D., Cellini A., Donati I., Pastore C., Onofrietti C., Spinelli F. (2020). Facing climate change: Application of microbial biostimulants to mitigate stress in horticultural crops. Agronomy.

[216] Law S.R., Mathes F., Paten A.M., Alexandre P.A., Regmi R., Reid C., Safarchi A., Shaktivesh S., Wang Y., Wilson A. (2024). Life at the borderlands: Microbiomes of interfaces critical to One Health. FEMS Microbiol. Rev..

[217] Seema N., Hamayun M., Hussain A., Shah M., Irshad M., Qadir M., Iqbal A., Alrefaei A.F., Ali S. (2023). Endophytic Fusarium proliferatum Reprogrammed Phytohormone Production and Antioxidant System of Oryza sativa under Drought Stress. Agronomy.

[218] Alfonzetti M., Doleac S., Mills C.H., Gallagher R.V., Tetu S. (2022). Characterizing effects of microbial biostimulants and whole-soil inoculums for native plant revegetation. Microorganisms.

[219] Caddell D.F., Deng S., Coleman-Derr D. (2019). Role of the plant root microbiome in abiotic stress tolerance. Seed Endophytes: Biology and Biotechnology.

[220] Fadiji A.E., Yadav A.N., Santoyo G., Babalola O.O. (2023). Understanding the plant-microbe interactions in environments exposed to abiotic stresses: An overview. Microbiol. Res..

[221] Ben Rejeb I., Pastor V., Mauch-Mani B. (2014). Plant responses to simultaneous biotic and abiotic stress: Molecular mechanisms. Plants.

[222] Bell J.C., Bound S.A., Buntain M. (2022). Biostimulants in agricultural and horticultural production. Hortic. Rev..

[223] Caradonia F., Battaglia V., Righi L., Pascali G., La Torre A. (2019). Plant biostimulant regulatory framework: Prospects in Europe and current situation at international level. J. Plant Growth Regul..

[224] Peter A.J., Amalraj E.L.D., Talluri V.R. (2020). Commercial aspects of biofertilizers and biostimulants development utilizing rhizosphere microbes: Global and Indian scenario. Rhizosphere Microbes: Soil and Plant Functions.

[225] Hajji-Hedfi L., Ibrahim D.S., Othmen S.B., El-Abeid S.E., Hlaoua W., Mosa M.A., Rhouma A., Riad S.N., Ghareeb S., El-Deriny M.M. (2025). Production of Microbial Biostimulants as a Response to the Modern Agricultural Need for Productivity and Plant Health. Microbial Biostimulants.

[226] Fadiji A.E., Babalola O.O., Santoyo G., Perazzolli M. (2021). The Potential Role of Microbial Biostimulants in the Amelioration of Climate Change-Associated Abiotic Stresses on Crops. Front. Microbiol..

[227] Bibi F., Rahman A. (2023). An overview of climate change impacts on agriculture and their mitigation strategies. Agriculture.

[228] Ikuyinminu E., Goni O., Langowski L., O’Connell S. (2023). Transcriptome, Biochemical and Phenotypic Analysis of the Effects of a Precision Engineered Biostimulant for Inducing Salinity Stress Tolerance in Tomato. Int. J. Mol. Sci..

[229] Lokko Y., Heijde M., Schebesta K., Scholtes P., Van Montagu M., Giacca M. (2018). Biotechnology and the bioeconomy-Towards inclusive and sustainable industrial development. New Biotechnol..

[230] Suman A., Govindasamy V., Ramakrishnan B., Aswini K., SaiPrasad J., Sharma P., Pathak D., Annapurna K. (2021). Microbial Community and Function-Based Synthetic Bioinoculants: A Perspective for Sustainable Agriculture. Front. Microbiol..

[231] Malik A., Mor V.S., Tokas J., Punia H., Malik S., Malik K., Sangwan S., Tomar S., Singh P., Singh N. (2020). Biostimulant-treated seedlings under sustainable agriculture: A global perspective facing climate change. Agronomy.

[232] Hamid B., Zaman M., Farooq S., Fatima S., Sayyed R., Baba Z., Sheikh T., Reddy M., El Enshasy H., Gafur A. (2021). Bacterial plant biostimulants: A sustainable way towards improving growth, productivity, and health of crops. Sustainability.

